# Emulsion-templated hierarchical silica monoliths functionalized with sulfonic acid for esterification and acetalization reactions

**DOI:** 10.1039/d6cy00824k

**Published:** 2026-09-21

**Authors:** Binhang Zhao, Pan Yang, Donald R. Inns, Angela Gondolini, Alessandra Sanson, Haifei Zhang

**Affiliations:** a Department of Chemistry, University of Liverpool Crown Street Liverpool L69 7ZD UK zhanghf@liverpool.ac.uk; b National Research Council of Italy, Institute of Science, Technology and Sustainability for Ceramics (CNR-ISSMC) Via Granarolo 64 48018 Faenza Italy

## Abstract

Efficient heterogeneous catalysts have been developed owing to their advantages in reusability and sustainability. However, conventional catalysts composed of fine or nanosized particles suffered from a high pressure drop and catalyst loss in fixed-bed or batch reactors, limiting their practical applications. In this work, macro–mesoporous hierarchical silica monoliths were fabricated *via* an emulsion-templating method with easily available silica colloids as building blocks. Post-grafting of –SH groups and subsequent oxidation with H_2_O_2_ generated hierarchically porous silica monoliths with sulfonic acid sites. The resulting H-(0.9) SO_3_-75%-silica exhibited an interconnected macroporous morphology with a pore volume of 2.67 cm^3^ g^−1^ and a surface area of 232.7 m^2^ g^−1^. NH_3_ temperature programmed desorption analysis confirmed the presence of acidic sites, while the sulfonic acid site density was estimated from the sulphur content determined by elemental analysis. The H-(0.9) SO_3_-75%-silica catalyst demonstrated high performance in both oleic acid-rich fatty acid feedstock esterification and glycerol acetalization. The free fatty acid conversion reached 95% at 90 °C in 6 h with a turnover frequency (TOF) of 32.1 h^−1^, while glycerol acetalization achieved 98.6% conversion and 98% solketal selectivity at 20 °C within 30 min (TOF = 1155 h^−1^). Moreover, the catalyst maintained high activity over five consecutive cycles, and its monolithic form allowed easy separation from the reaction system, indicating its potential suitability for practical continuous or fixed-bed applications.

## Abstract 2

Efficient heterogeneous catalysts with interconnected macropores have been developed for esterification and acetalization reactions with high reusability and sustainability.

## Graphical abstract

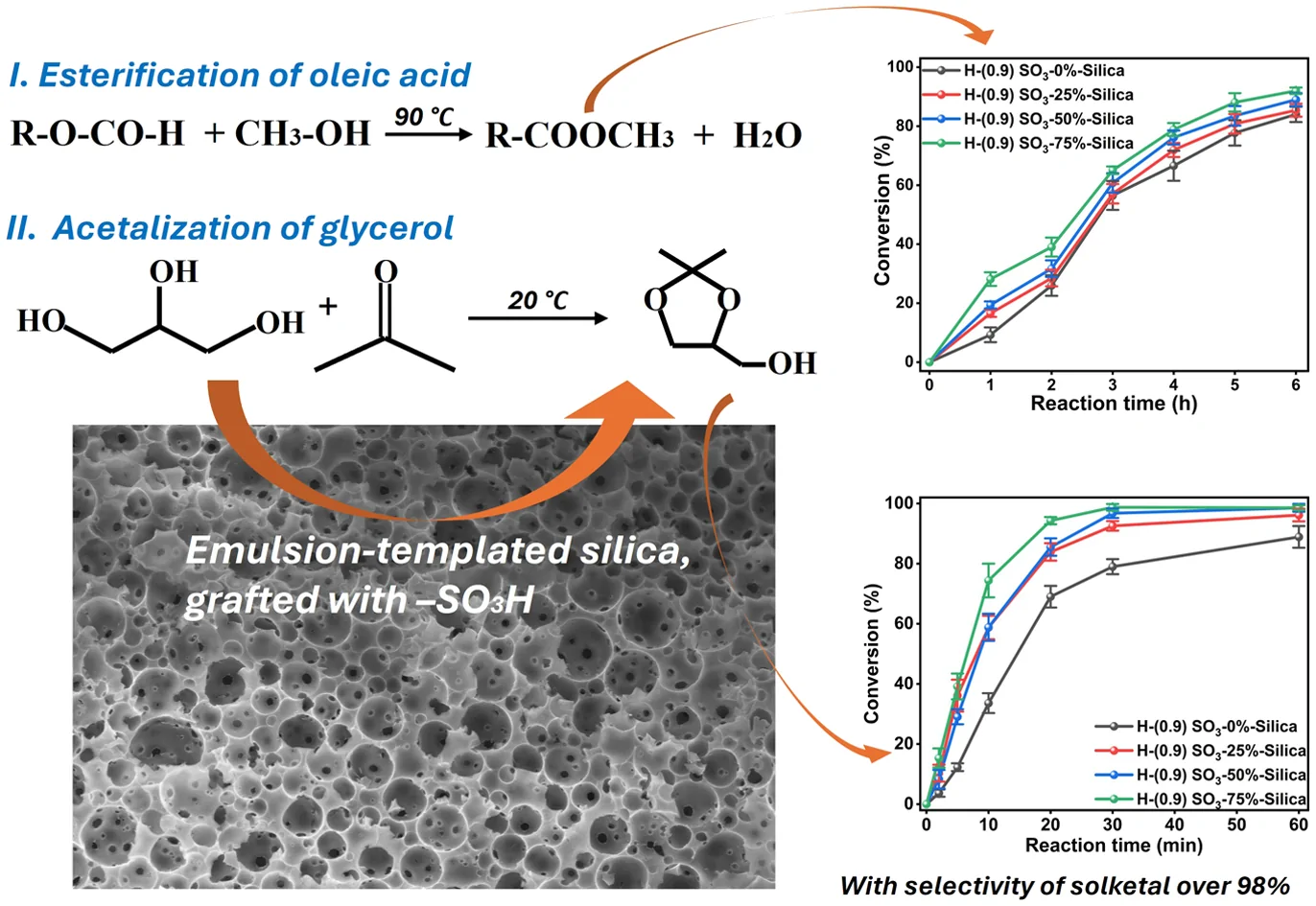

## 1. Introduction

Catalysis plays a crucial role in both chemical and biological transformations, driving over 90% of chemical manufacturing processes, including the production of fuels, fine chemicals, and pharmaceuticals.^1,2^ Catalysts are generally classified as homogeneous or heterogeneous.

Homogeneous catalysts typically exhibit superior catalytic activity due to their well-defined active sites and excellent solubility in the reaction medium. However, their separation from the reaction mixture is often difficult, resulting in poor recyclability, increased environmental burden, higher operational costs, and equipment corrosion. These limitations restrict their widespread industrial applications.^3^ In contrast, heterogeneous catalysts are more compatible with the principles of green chemistry, as they reduce environmental pollution, facilitate catalyst recovery, and minimize equipment corrosion.^4^ Consequently, heterogeneous catalysis has attracted significant attention, particularly for industrial applications.

Catalyst supports play an important role in catalytic performance. Catalyst supports interact with active components or surface functional groups, influencing their dispersion, stability, and catalytic performance.^5–7^ While the support material may not directly participate in the catalytic reaction, its physicochemical properties, including hydrophobicity, hydrophilicity, and porosity, play an important role in enhancing overall catalytic efficiency.^8^ Additionally, catalyst supports contribute to the physical form, texture, and mechanical stability of the catalyst; both the surface functional groups and physical characteristics of the support material significantly impact catalytic performance.^2,9^ Among the commonly used catalyst supports including alumina, silica, and carbon materials, silica (SiO_2_) serves as an excellent support due to its chemical inertness, high thermal stability, cost-effectiveness, and facile surface group functionalisation.^2^ Furthermore, its tuneable porosity is a key characteristic for the catalytic performance by influencing the diffusion of reactants and accessibility of active sites.^10^ Liu *et al.* introduced methyl groups adjacent to copper nanoparticles on the silica surface, resulting in a silica-supported copper catalyst that exhibited exceptional durability during the hydrogenation of dimethyl oxalate.^11^ Kim *et al.* reported the synthesis of Pd-supported KCC-1 (wrinkled silica), which demonstrated excellent catalytic performance in the conversion of biomass-derived furfural (FF) to tetrahydrofurfuryl alcohol (THFAL), achieving 100% FF conversion and 85% THFAL selectivity within 6 hours at 50 °C under 20 bar H_2_.^12^ Mazarío *et al.* reported the preparation of silica-supported CuO catalysts that were highly active and selective for acetol production *via* glycerol dehydration in a continuous-flow fixed-bed reactor.^13^

Macropores are often introduced into catalyst materials to enhance mass transport in heterogeneous catalysis.^14^ Emulsion templating and ice templating are two commonly used approaches for the preparation of macroporous materials.^15,16^ Emulsions are heterogeneous mixtures of two immiscible liquids, where one is dispersed as small droplets in the other, typically stabilized by surfactants or solid particles.^17^ It is possible to tune the pore size and porosity of macroporous materials by varying the droplet size and the number of droplets in an emulsion. Kapadnis *et al.* prepared hollow silica aerogel particles with precise control over their characteristics using an oil-in-water-in-oil double emulsion method. The fabricated silica aerogel particles have potential applications in drug delivery, catalysis and insulation.^18^ Mattich *et al.* synthesised macroporous silica glass monoliths through emulsion templates prepared by step emulsification in microfluidic devices. The synthesised silica glass monoliths with unique cellular architectures may be used as filters, separation membranes, or catalyst supports.^19^ Cho *et al.* fabricated silica aerogels using attractive silica nanolace (ASNL)-armoured Pickering emulsion templates, yielding ultralight, mechanically robust, and thermally insulating materials potentially suitable for building services, energy-saving technologies, and aerospace applications.^20^ These approaches highlight the versatility of emulsion templating in controlling pore size and framework structure. Owing to their high macropore density, these materials are promising catalyst supports that disperse acid sites effectively and lessen diffusion constraints.

Biodiesel is seen as a potential alternative to fossil fuels and it offers multiple advantages over conventional fuels, including the use of renewable feedstocks, reduced greenhouse gas emissions, cleaner combustion, biodegradability, and compatibility with existing diesel engines.^21^ Transesterification is the primary chemical reaction used in biodiesel. Although both bases and acids can promote this reaction, base-catalysed transesterification typically exhibits significantly higher catalytic efficiency and is therefore commonly employed in industrial processes.^22,23^ However, some non-edible oils contain relatively high free fatty acids (FFAs), which can react with a basic catalyst to produce soap during transesterification. Soap formation increases the viscosity of the product and makes separation from the reaction mixture very difficult.^24^ Therefore, a prior esterification of FFAs and alcohol is essential to prevent soap formation and simultaneous conversion of FFAs into biodiesel (Scheme 1a).^25^ The transesterification of triglycerides and methanol yields biodiesel and glycerol as the main products. With the increasing global production of biodiesel, glycerol has become an oversupplied by-product. Although glycerol is not directly applicable as fuel, it can be transformed into a range of valuable chemicals. Among these, the acetalization of glycerol with acetone to form solketal is a promising approach (Scheme 1b).^26^ Solketal can serve as a fuel additive, improving the cold-flow properties and reducing particulate emissions of liquid transportation fuels. Furthermore, its addition enhances oxidation stability, suppresses gum formation, and increases the octane number of gasoline blends.^27^ Acetalization of glycerol and acetone commonly yields six-membered 1,3-dioxane (acetal) as a by-product, in addition to the five-membered solketal, with both pathways catalysed by an acid.

**Scheme 1:**
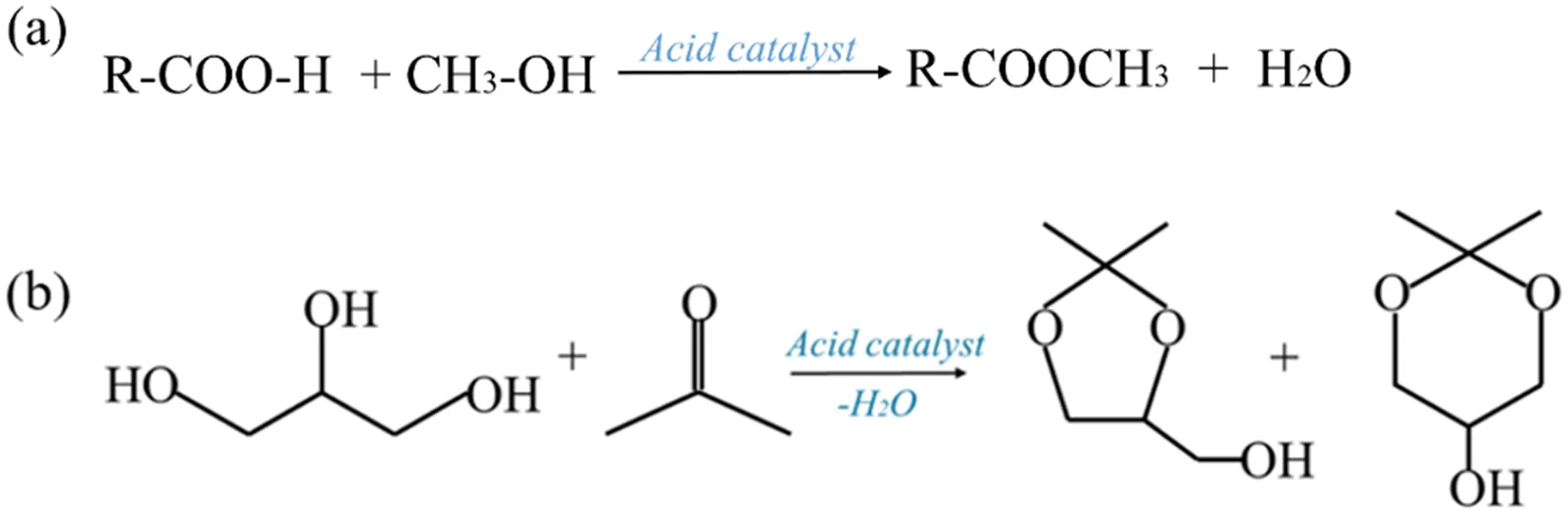
(a) Esterification of fatty acids and methanol. (b) Acetalization of glycerol and acetone.

A variety of solid acid catalysts have been developed for esterification and acetalization reactions. Lu *et al.* grafted an ionic liquid onto NH_2_-UiO-66 and applied the resulting material to the esterification of oleic acid, showing excellent catalytic activity and good recyclability.^28^ Zhao *et al.* immobilized H_3_PMo_12_O_40_ on a covalent organic framework (COF) material, and the obtained catalysts exhibited high activity and stable reusability in the esterification reactions.^29^ Santos-Vieira *et al.* prepared a Gd^3+^ based coordination polymer using nitrilo (trimethylphosphonic acid) as the ligand, which showed good catalytic performance and recyclability in acetalization of glycerol.^30^ Malakar *et al.* reported a highly acidic microporous hybrid titanium phosphate material formed by incorporating 5-aminosalicylic acid into the titanium phosphate framework. This catalyst efficiently promoted glycerol acetalization with over 99% selectivity of solketal.^31^ Despite their promising catalytic activities, all of these catalysts lack macropores, which limits the mass transport of reaction molecules, particularly for relatively large molecules in a viscous reaction system. Furthermore, these catalysts are usually prepared in the form of small particles, making recovery and reuse practically difficult, and their high preparation cost limits their suitability for large-scale applications. In our previous work, sulfonated ZSM-5 monoliths were synthesized and evaluated as a solid acid catalyst,^32^ but their insufficient macropores and mesopores restricted mass transport and limited catalytic efficiency.

Compared with sulfonated mesoporous silica, silica nanoparticles and aerogels, the emulsion-templated hierarchical sulfonated silica monoliths developed in this work offer several advantages. Fine mesoporous silica particles and silica nanoparticles can suffer from difficult recovery in liquid-phase reactions and may induce a high pressure drop or blockage in fixed-bed or microreactor systems, while silica aerogels often require costly drying processes and show poor mechanical robustness.^33–35^ In contrast, monolithic silica with interconnected macro–mesoporous structures can facilitate fluid transport while reducing the pressure drop in packed-bed reactors. Its integrated monolithic architecture also makes it suitable for use in micro/mesoreactors and other continuous-flow systems.^36^ The novelty of this work lies in the integration of commercial colloidal silica, an emulsion-templated hierarchical monolithic architecture and post-synthetic functionalisation of sulfonic acid. The use of commercial silica colloids and readily available reagents also provides a practical basis for lower-cost preparation, while the monolithic structure enables facile separation and potential application in packed-bed or continuous-flow catalytic systems.

In this study, a hierarchical macro–mesoporous silica monolith was synthesized *via* oil-in-water (O/W) emulsion templating and simple gelation by a pH change in the continuous phase. Commercially available HS-30 silica colloids were used as the building blocks. The prepared porous silica materials contain emulsion-templated macropores and mesopores & high surface areas generated from agglomerated silica colloids. (3-Mercaptopropyl) trimethoxysilane (3-MPTMS) was subsequently grafted onto the silica framework, and oxidation of thiol groups into sulfonic acid groups under mild conditions with H_2_O_2_ significantly increased the density of acid sites on the material. The resulting catalyst exhibited excellent catalytic performance in both esterification and acetalization reactions along with good reusability, and its monolithic form allowed easy recovery from the reaction system.

## 2. Experimental

### 2.1 Chemicals and reagents

HS-30 colloidal silica (30 wt% suspension in water, pH 9 and 10), sodium dodecyl sulfate (SDS, ≥99%), (3-mercaptopropyl)trimethoxysilane (3-MPTMS), polyvinyl alcohol (PVA, MW 9000–10 000, 80% hydrolysis), tetraethyl orthosilicate (98%, TEOS), oleic acid-rich free fatty acid (65–88%), phenolphthalein, oxalic acid (98%), and sodium hydroxide (≥98%) were purchased from Merck. Cyclohexane (≥99%), nitric acid (70%), toluene (≥99.8%), methanol (≥99%), acetone (≥99%), propan-2-ol (≥99.5%), diethyl ether (≥99%), ethyl acetate (≥99.5%), potassium hydroxide (86.5%), and absolute ethanol were bought from Fisher Scientific. The hydrogen peroxide solution (35%) was provided by Avantor Sciences.

### 2.2 Preparation of emulsion-templated silica monoliths

To prepare the emulsion, 0.25 g of PVA was dissolved in 5 mL of de-ionized (DI) water under stirring at 600 rpm for 20 minutes at room temperature. 5 mL of HS-30 colloidal silica suspension was subsequently added to the PVA solution. Then, 0.5 g of SDS was dissolved in the mixture. After complete dissolution, 30 mL of cyclohexane was added into the solution dropwise under stirring at 600 rpm, followed by an additional 20 min of stirring. To investigate the effect of ratio of oil and water, different volumes of cyclohexane (0 mL, 3.33 mL, 10 mL and 30 mL) were used, corresponding to 0%, 25%, 50% and 75% of the total emulsion volume, respectively. 2.5 mL of pre-hydrolysed acidic TEOS sol was added to the emulsion. The gelation of the emulsion continuous phase occurred rapidly due to the basic nature of HS-30 dispersion, resulting in the formation of silica monoliths. After allowing the gelation process to complete by leaving the sample in an oven at 50 °C for 8 h with a lid, the sample was finally placed in a vacuum oven at 70 °C overnight to remove residual cyclohexane and water.

### 2.3 Preparation of the TEOS sol used to gel HS-30 colloids

0.08 g of oxalic acid was dissolved in 14.4 mL of DI water under stirring at 500 rpm. 44.7 mL of TEOS was subsequently added to the solution at room temperature under stirring at 500 rpm for 1 h to form a homogeneous sol. The homogeneous TEOS sol was stored in a freezer at −20 °C for further use. The molar ratio was 1(TEOS) : 4(H_2_O) : 0.0044 (oxalic acid).

### 2.4 Preparation of non-emulsion-templated silica materials as controls

This included the direct gelation of the water-based TEOS sol and HS-30 colloidal dispersion, without adding cyclohexane to form emulsions, by placing them in an oven at 50 °C for 24 h. These control samples were: (a) a TEOS monolith from the TEOS sol formed in section 2.3, (b) a TEOS + SDS monolith (typically, 1.44 g of SDS was added to 10 mL of TEOS sol before gelation) and (c) a silica monolith formed by direct evaporation of an HS-30 colloidal suspension in air.

### 2.5 Calcination

Calcination of the silica materials was carried out in a Carbolite furnace in air. The temperature was increased to 250 °C at a rate of 1 °C min^−1^ and held for 1 h. Subsequently, the temperature was raised to 550 °C at the same heating rate and maintained for 5 h. Finally, the sample was cooled to room temperature at a rate of 3 °C min^−1^.

### 2.6 Preparation of sulfonated silica monoliths

To enhance the density of silanol groups in the monoliths, the calcined silica materials (1.0 g) were treated in 0.1 M HNO_3_ (30 mL) at 100 °C for 24 h using a round-bottom flask fitted with a condenser. After the acid treatment, the monoliths were used directly for subsequent modification with 3-MPTMS without washing or drying in order to retain the water and residual nitric acid inside the silica framework. This was because the comparison study on washing and drying of the acid-treated silica samples indicated a detrimental effect on 3-MPTMS grafting onto silica.

The acid treated monolith was added into the solution containing 3-MPTMS (0.9 g) and toluene (30 mL) in a round-bottom flask, which was equipped with a waterless condenser and then heated in an oil bath at 110 °C for 24 h to complete the grafting process. Two different mass ratios of monolith to 3-MPTMS (1 : 0.9 and 1 : 2.7) were employed. The obtained monoliths were washed three times with toluene to remove the residual 3-MPTMS. After washing, the samples were placed in a vacuum oven and dried at 70 °C overnight.

1 g of dried 3-MPTMS-treated monoliths were oxidized with 60 mL of hydrogen peroxide at room temperature for 24 h.^32^ The fabrication procedure for the sulfonated hierarchical silica monoliths is illustrated in Scheme 2. After oxidation, the samples were washed three times with DI water and dried in a vacuum oven overnight at 70 °C before being employed as catalysts.

**Scheme 2:**
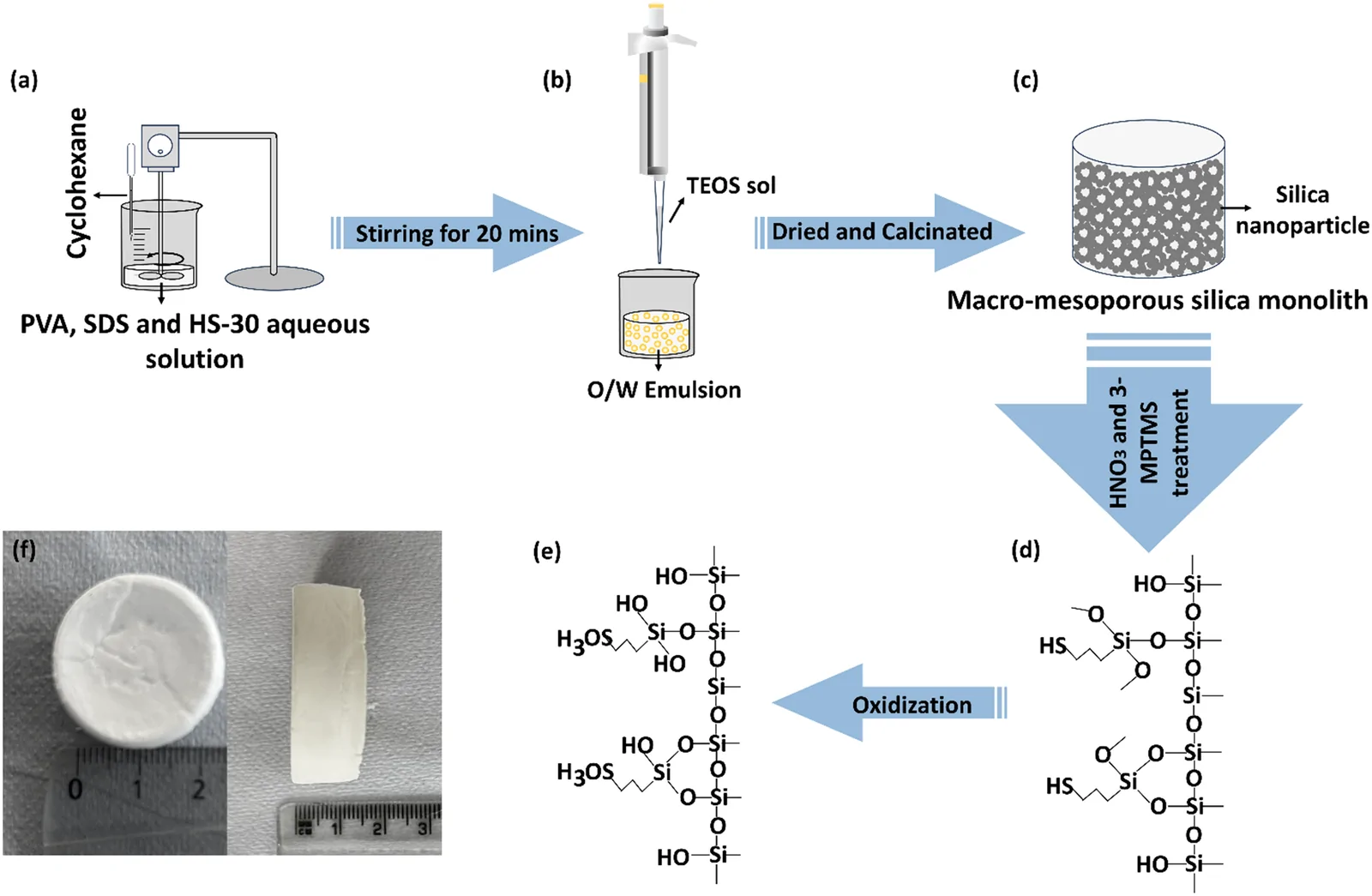
The fabrication process of hierarchical sulfonated silica monoliths. (a) An oil-in-water (O/W) emulsion formed by adding cyclohexane into the aqueous phase containing HS-30 colloids, surfactant SDS, and stabilizer PVA. (b) The pre-hydrolysed TEOS sol underwent rapid gelation upon addition into the continuous phase of the emulsion, locking in the emulsion-templated structure. (c) Following a drying process and a calcination process to remove PVA and SDS, a silica monolith containing mesopores and emulsion-templated macropores is produced. (d) The resulting silica monolith is then treated with HNO_3_ to enhance the density of surface silanol groups, followed by grafting with 3-MPTMS. (e) Subsequent oxidation with H_2_O_2_ leads to the formation of –SO_3_H groups within the hierarchically porous silica monolith, as shown in (f).

The fabricated silica monoliths were denoted as H-(*A*) SO_3_-*B*-silica, where H stands for treatment with HNO_3_, *A* indicates the mass ratio of 3-MPTMS to monoliths, and *B* specifies the different ratios of cyclohexane and total emulsion volume. For example, 1 g of silica monolith prepared from the 75% emulsion was first treated with HNO_3_, followed by grafting with 0.9 g of 3-MPTMS. The resulting sample was denoted as H-(0.9) SO_3_-75%-silica. When the monoliths were not treated with HNO_3_ but underwent the same grafting procedure, the sample was denoted as (0.9) SO_3_-75%-silica. Monoliths prepared under identical conditions without the sulfonation step were denoted as H-75%-silica.

### 2.7 Catalyst characterization

The porous structure of the silica monoliths was characterized using a scanning electron microscope (Hitachi S-4800 SEM) operated at an accelerating voltage of 10 kV, using a secondary electron detector. Small fragments of the monolith samples were mounted on double-sided adhesive carbon tape attached to an aluminium stub and subsequently coated with a thin gold layer using a sputter coater operated at 30 mA for 60 seconds to enhance conductivity prior to imaging.

Fourier transform infrared (FTIR) spectroscopy was used to identify functional groups, using a Bruker Vertex 70 spectrometer. The spectra were collected with 128 scans at a resolution of 4 cm^−1^ in the range of 4500–400 cm^−1^. Elemental compositions, namely, carbon, hydrogen, and sulphur, were determined with a CHNS elemental analyser (Elementar Vario Micro Cube).

The quantification of macropores by macropore volumes and bulk density was performed by mercury intrusion porosimetry (MIP, Thermofinnigan Pascal 240). The dry silica samples (∼20–30 mg) were measured with a pressure increase rate of 4–8 MPa min^−1^ up to a maximum pressure of 200 MPa.

Nitrogen sorption isotherms were recorded on a Micromeritics 3-Flex 3500 gas adsorption analyser. Prior to analysis, the samples were degassed under vacuum at 100 °C for 15 h. Specific surface areas were calculated by applying the Brunauer–Emmett–Teller (BET) model. Pore size distribution was determined using the non-local density functional theory (NLDFT) method, and mesopore volume was evaluated using the Barrett–Joyner–Halenda (BJH) approach.

X-ray photoelectron spectroscopy (XPS) measurements were carried out at the EPSRC National Facility for XPS (HarwellXPS) using a Kratos Axis SUPRA spectrometer equipped with a monochromated Al Kα X-ray source (700 × 300 μm).

Thermogravimetric analysis (TGA) was conducted on a Netzsch TG 209 F1 Libra instrument to assess the thermal stability and decomposition behaviour of the monoliths. The temperature was ramped from 30 °C to 800 °C at a rate of 20 °C min^−1^ under a nitrogen atmosphere.

Ammonia temperature-programmed desorption (NH_3_-TPD) measurements were conducted with a Micromeritics AutoChem 2920 II chemisorption analyser equipped with a thermal conductivity detector (TCD).

### 2.8 Esterification of oleic acid-rich fatty acid feedstock and methanol

The esterification reactions were conducted in 20 mL sealed autoclave vessels under autogenous pressure, without the introduction of any external gas. The autoclaves were placed in a preheated oven to maintain the desired reaction temperature. A solution containing 3.17 mL of oleic acid-rich free fatty acid and 4.05 mL of methanol was pre-mixed thoroughly before introducing the catalyst. Catalyst loadings equivalent to 1 wt%, 3 wt%, and 5 wt% of the mass of oleic acid were evaluated. To examine the effect of temperature, the reactions were performed at 90 °C, 120 °C, and 150 °C. Upon completion, the esterification mixtures were concentrated using a rotary evaporator (Buchi B-480) at 50 °C for 6 h to remove water and unreacted methanol. Error bars represent the standard deviation from three independent catalytic experiments.

The oleic acid used in this study contained other monocarboxylic acids such as linoleic acid, palmitic acid and stearic acid. The conversion was calculated based on the total free fatty acid instead of oleic acid only, using the acid values determined by titration. Direct quantification by gas chromatography (GC) analysis for each acid would make the calculation of the conversion of fatty acids in the sample very complicated. The acid values before and after the reaction were determined by titration with a 0.2 M KOH solution, using phenolphthalein as an indicator, in accordance with the established procedure reported in the literature.^37^ The acid value was calculated using the following formula:
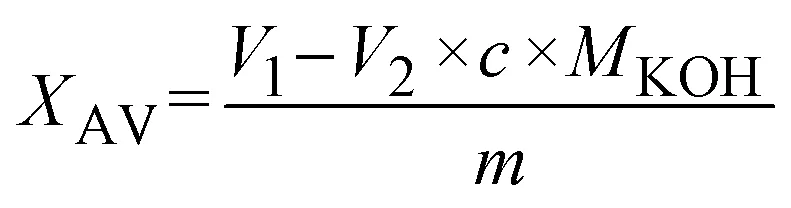
where *X*_AV_ (mg g^−1^) represents the acid value, *V*_1_ and *V*_2_ (mL) denote the initial and final volumes of KOH, *c* (mmol mL^−1^) is the concentration of KOH, *M*_KOH_ (mg mmol^−1^) represents the molecular weight of KOH, and *m* (g) indicates the mass of evaporated esterification product.

The conversion of free fatty acid was assessed based on the change in acid value, and calculated using the following equation:^38^
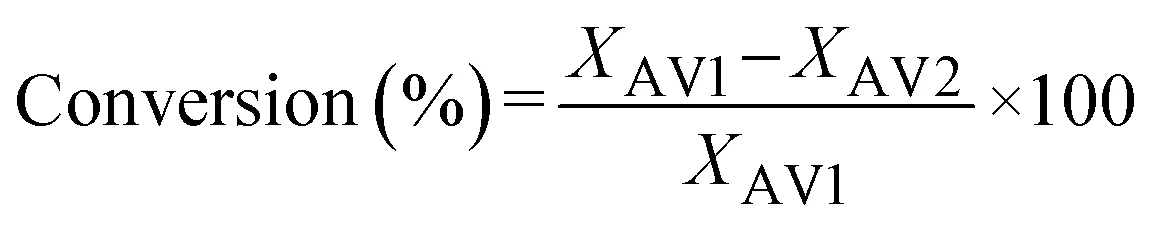
where *X*_AV1_ (mg g^−1^) refers to the initial acid value of the fatty acid feedstock and *X*_AV2_ (mg g^−1^) represents the acid value of the final esterification product following evaporation.

Since the commercial oleic acid-rich free fatty acid feedstock contains a mixture of monocarboxylic fatty acids, *N* (mmol) was defined as the initial amount of total free fatty acid equivalents. Accordingly, one mole of titratable carboxylic acid was assumed to correspond to one mole of free fatty acid equivalent, and *N* was calculated from the initial acid value of the oleic acid-rich free fatty acid feedstock using the following equation:
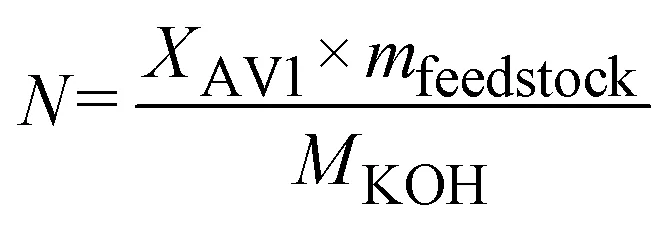
where *X*_AV1_ (mg g^−1^) is the initial acid value of the free fatty acid feedstock, *m*_feedstock_ (g) is the mass of free fatty acid feedstock used in the reaction, *M*_KOH_ (mg mmol^−1^) represents the molecular weight of KOH. Therefore, *N* is expressed in mmol.

The turnover frequency (TOF, h^−1^) was calculated based on the free fatty acid conversion during the first 1 h of reaction using the followingequation:^39^
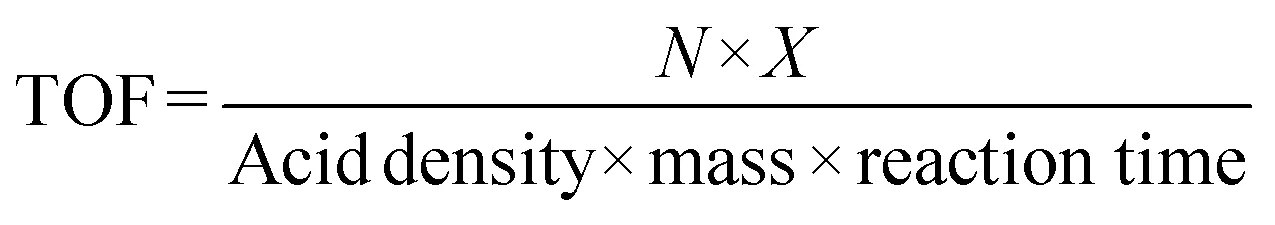
Here, TOF (h^−1^) represents the number of catalytic turnovers per active site per hour. *N* (mmol) denotes the initial amount of total free fatty acid equivalents, *X* (%) represents the conversion rate of free fatty acid. Meanwhile acid density (mmol g^−1^) was determined *via* CHNS elemental analysis. The catalyst amount is expressed as its mass (g), and the reaction time is given in hours (h).

It should be noted that the acid density used for TOF calculation was evaluated from the total sulphur content determined by CHNS elemental analysis. This value may include sulfonic acid groups that are not fully accessible to reactants. Therefore, the number of accessible acid sites may be overestimated, and the calculated TOF values may underestimate the intrinsic activity of the accessible acid sites. All esterification experiments were carried out three times, and the error bars represent the standard deviation.

H-(0.9) SO_3_-50%-silica was selected for the reusability study because it offered a better balance between catalytic activity and mechanical stability during repeated handling, despite the higher catalytic performance of H-(0.9) SO_3_-75%-silica. The reusability of the fabricated catalyst was evaluated over five consecutive cycles. After each catalytic cycle, the used H-(0.9) SO_3_-50%-silica was washed three times by soaking in ethyl acetate for 1 h per wash to remove residual fatty acids and ester products. The washed catalyst was then dried overnight in a vacuum oven at 70 °C before reuse.

### 2.9 Acetalization of glycerol

The acetalization reaction was carried out in a 25 mL double-necked round-bottom flask equipped with a condenser and immersed in an oil bath under continuous stirring at 600 rpm. A mixture of 1.25 g glycerol and 10.5 mL acetone was added to the flask, which was then heated to the target temperature. After preheating for 5 min, the catalyst was introduced in various loadings of 1 wt%, 3 wt%, and 5 wt%, based on the mass of glycerol. To investigate the influence of temperature, the reactions were conducted at 20 °C and 40 °C. Aliquots were withdrawn at different reaction times and analysed by GC-MS to monitor conversion and selectivity. The glycerol conversion and solketal selectivity were calculated using the below formula:
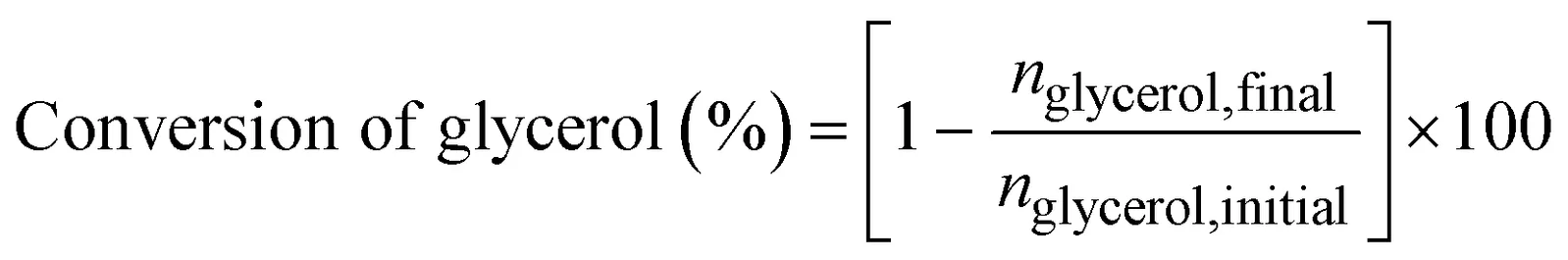

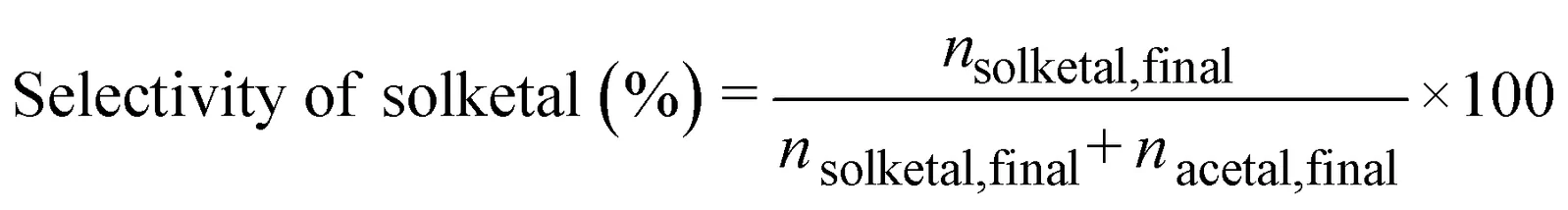
where *n* (mol) represents the amount in moles.

The glycerol-based carbon balance was determined by quantifying liquid-phase components using GC-FID. As glycerol is converted predominantly to solketal and its acetal isomer with a 1 : 1 stoichiometry with respect to glycerol. The glycerol-based carbon content can be calculated according to the following equation:^40^
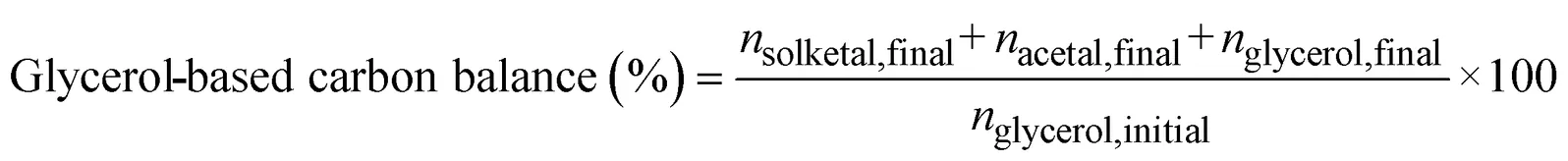
where *n* (mol) represents the amount in moles.

The TOF for the acetalization reaction was calculated using the same equation as applied for the esterification reaction above, based on the glycerol conversion during the initial 2 min of reaction. The limitation associated with using total sulphur-derived acid density also applies to this calculation. All acetalization experiments were repeated three times, and the error bars show the standard deviations.

The reusability of H-(0.9) SO_3_-50%-silica was also assessed in glycerol acetalization over five consecutive cycles. After each cycle, the spent catalyst was washed three times by soaking in methanol for 1 h per wash to remove the residual glycerol and solketal, and then dried overnight in a vacuum oven at 70 °C for the next cycle of test, which was conducted under the same conditions as fresh catalyst testing.

## 3. Results and discussion

### 3.1 Catalyst structure and surface properties

#### 3.1.1 Morphology of the catalysts

SEM was employed to examine the morphology of the fabricated sulfonated silica monoliths, as shown in Fig. 1. The formation of droplet-templated macropores is attributed to the droplets of cyclohexane in the O/W emulsion during synthesis. The addition of the TEOS sol (pre-formed by hydrolysis of TEOS under acidic conditions) led to rapid gelation of the continuous phase of the emulsion, which solidified the structure and preserved the emulsion-templated morphology. HS-30 was a clear and stable suspension of silica nanoparticles (diameter ∼15 nm, 30 wt%), with the suspension pH = 9.8.^41^ Aggregation of HS-30 silica nanoparticles could be initiated when the pH of the suspension was reduced. The interstices of aggregated silica nanoparticles generated mesopores and high surface area. The sol–gel process with the TEOS sol could glue the silica nanoparticles together and enhance mechanical stability of the resulting material. These macropores were retained with minimal shrinkage after the removal of both cyclohexane and water in a vacuum oven. A clear trend was observed: with the increasing volume ratio of cyclohexane in the emulsions, the quantity and connectivity of macropores in the resulting emulsion-templated materials increased (Fig. 1). The sample with 0% cyclohexane showed no visible macropores, while the 25% sample exhibited a limited number of isolated pores. At 50%, a greater number of macropores were present, and at 75%, a continuous and well-connected macroporous network was produced. The interconnected macropores can significantly improve the diffusion of reactants and products, and enhance the accessibility of active sites, thereby improving the catalytic performance relative to non-porous structures.^42^ The preliminary trials showed that it was possible to obtain emulsion-templated materials with >75% of the internal phase (*i.e.*, cyclohexane) to provide higher interconnected macroporosity. However, it was noted that those materials were fragile after calcination and exhibited very poor mechanical stability, which made them difficult to handle for the catalytic reactions. Therefore, 75% cyclohexane in the emulsions was selected as the highest volume ratio of internal phase in this study.

**Fig. 1:**
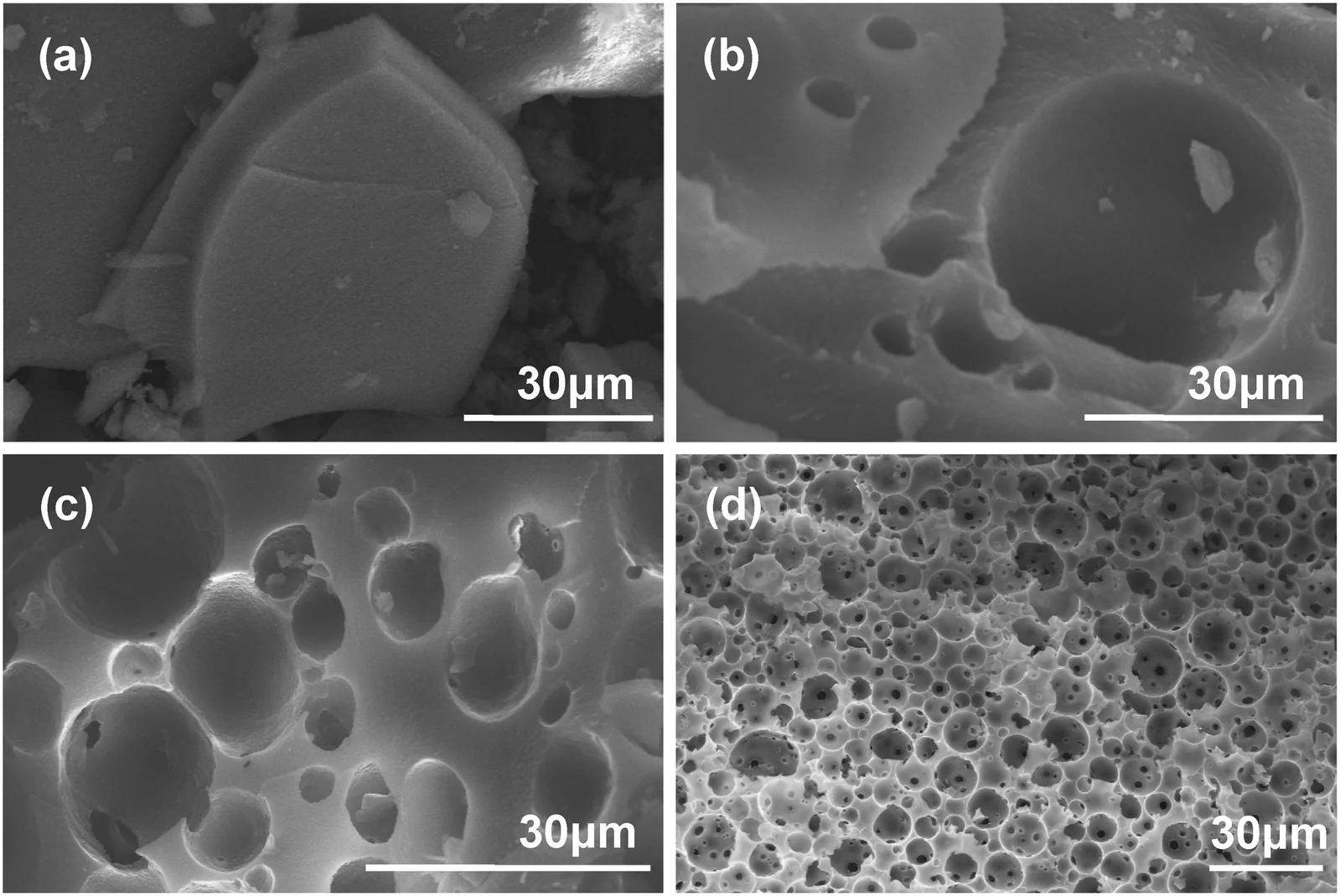
SEM images of hierarchical sulfonated silica monoliths prepared from emulsions with varying volume ratio of cyclohexane in the O/W emulsions. (a) H-(0.9) SO_3_-0%-silica, from aqueous dispersion without cyclohexane. (b) H-(0.9) SO_3_-25%-silica, from the emulsion with 25 v/v% cyclohexane. (c) H-(0.9) SO_3_-50 v/v%-silica, from the emulsion with 50% cyclohexane. (d) H-(0.9) SO_3_-75%-silica, from the emulsion with 75 v/v% cyclohexane.

#### 3.1.2 Macroporosity of emulsion-templated silica monoliths

Emulsion-templated macropores can be more effectively characterised by mercury intrusion porosimetry (MIP). The MIP measurements were conducted on the silica monoliths after calcination and acid treatment, prepared from the emulsions with different volume ratios of droplet phase (cyclohexane). A sulfonated silica monolith prepared from 75 v/v% emulsion was also analysed. Table 1 shows the intrusion macropore volume and bulk density of these silica monoliths. Fig. 2a illustrates the profiles of cumulative mercury intrusion volume with increasing pressure (0–200 MPa). It can be seen that the macropore volume increased with an increasing volume ratio of cyclohexane in the emulsions, reaching 3.28 cm^3^ g^−1^ at 75% emulsion *versus* 0.88 cm^3^ g^−1^ at 0% emulsion, confirming that emulsion-templating effectively generates macroporosity. The pore-size distributions (Fig. 2b) show a pronounced peak at 1–2 μm for H-75%-silica, indicating the highly interconnected nature of the macropores with relatively uniform interconnected window sizes connecting the macropores, whereas the H-50%-silica only exhibits flat, broad peaks at larger diameters (∼5–50 μm). For the samples prepared from 0% and 25% emulsions, the peaks are negligible. Consistent with this observation, H-75%-silica displays the lowest bulk density (0.17 g cm^−3^, Table 1), indicative of the highest overall porosity.

**Table 1:** The intrusion volume and bulk density of emulsion-templated silica materials, measured by mercury intrusion porosimetry

Catalyst	Intrusion pore volume (cm^3^ g^−1^)	Bulk density (g cm^−3^)
H-0%-silica	0.88	0.57
H-25%-silica	0.92	0.54
H-50%-silica	1.64	0.30
H-75%-silica	3.28	0.17
H-(0.9) SO_3_-75%-silica	2.67	0.23

**Fig. 2:**
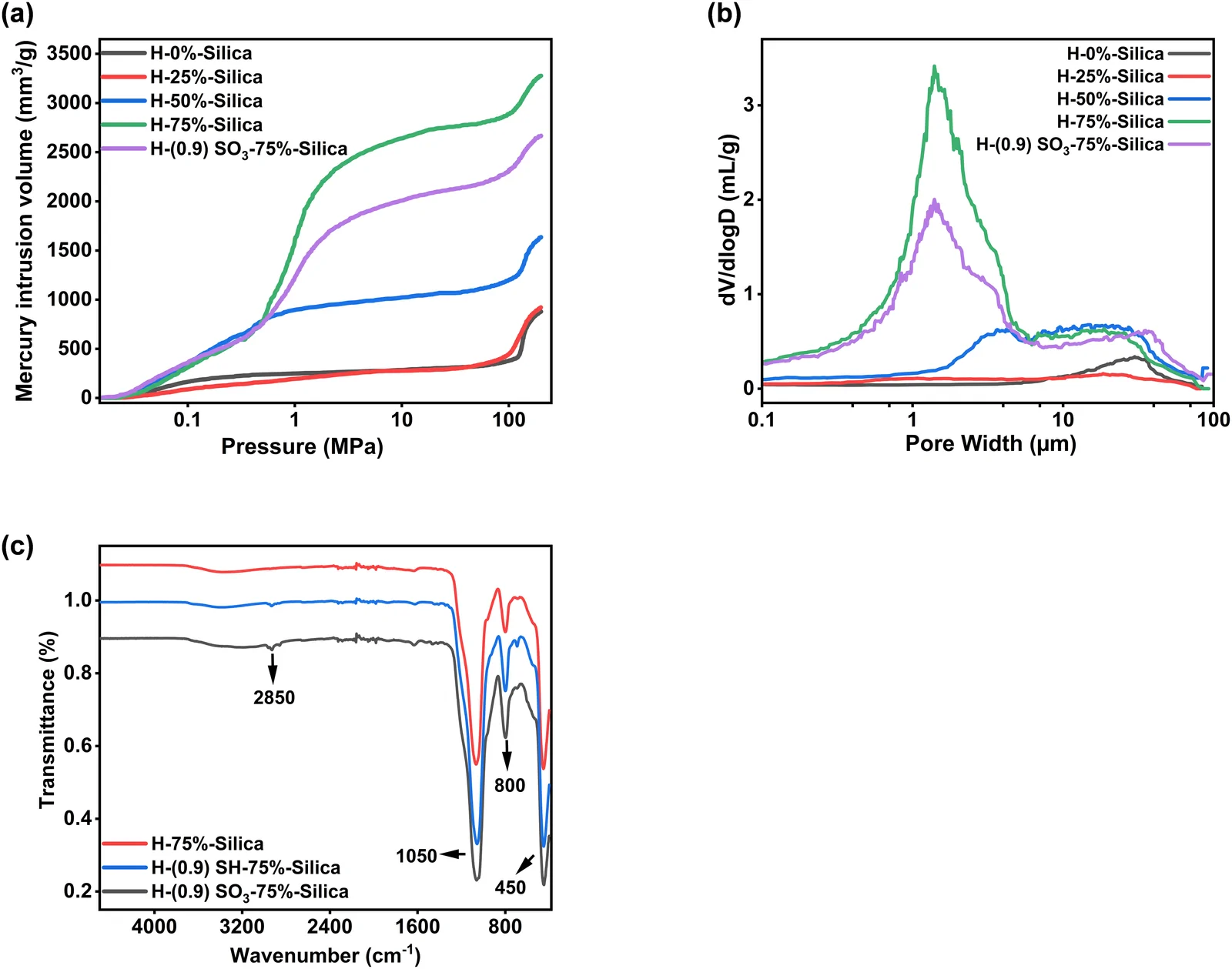
(a) Mercury intrusion porosimetry curves of silica monoliths. (b) Macropore size distribution analysis of silica monoliths using mercury intrusion porosimetry. (c) FT-IR spectrum of H-75%-silica, H-(0.9) SH-75%-silica, and H-(0.9) SO_3_-75%-silica.

After sulfonation, the macropore volume decreased slightly from 3.28 to 2.67 cm^3^ g^−1^, accompanied by an increase in bulk density from 0.17 to 0.23 g cm^−3^. The pore size distributions are comparable for these two silica samples but with a reduced intensity after the modification with –SO_3_H groups (Fig. 2b), indicating partial blockage or narrowing of the pore windows connecting the macropores after the modification process.

#### 3.1.3 FT-IR analysis

Fourier-transform infrared spectroscopy (FT-IR) was employed to characterize the functional groups present on the silica monoliths (Fig. 2c). For the emulsion-templated sample prepared from the emulsion with the 75 v/v% internal phase, after calcination, broad absorption bands observed between 3500 and 3300 cm^−1^ are attributed to O–H stretching vibrations, indicating the presence of surface hydroxyl groups. A prominent band showed around 1050 cm^−1^ corresponds to the asymmetric stretching and bending vibrations of the Si–O–Si framework, while the weaker peak near 800 cm^−1^ is assigned to the symmetric bending mode of Si–O–Si.^43^ Additionally, the band at approximately 462 cm^−1^ is associated with O–Si–O bending vibrations, further confirming the integrity of the silica network.

For the sulfonated samples, characteristic S=O stretching bands typically observed near 1050 and 1170 cm^−1^ are overlapped by the strong Si–O–Si signals and thus are not distinctly identifiable in the spectra.^44^ However, the appearance of aliphatic sp^3^ C–H stretching bands (–CH_2_–/–CH_3_) around 2850 cm^−1^ in both the 3-MPTMS-grafted and sulfonated samples confirms the successful grafting of 3-MPTMS onto the silica monolith surface, but does not provide direct evidence for the oxidation state of sulphur.^45^

#### 3.1.4 Surface area and mesoporosity

The fabricated samples were characterised by N_2_ sorption to evaluate the mesoporosity and specific surface areas. Table 2 summarizes the surface properties of sulfonated samples. Among the non-emulsion-templated samples, H-(0.9) SO_3_-TEOS + SDS monolith, H-(0.9) SO_3_-HS-30 and H-(0.9) SO_3_-0%-silica exhibited relatively high surface areas of 200.9 m^2^ g^−1^, 117.3 m^2^ g^−1^ and 243.2 m^2^ g^−1^, respectively. In contrast, the H-(0.9) SO_3_-TEOS monolith showed a significantly lower surface area of 9.4 m^2^ g^−1^. The considerable increase in surface area for H-(0.9) SO_3_-TEOS + SDS monolith is attributed to the presence of SDS micelles in the TEOS sol, which act as soft templates.^46^ Upon calcination, the removal of these micelles resulted in the formation of pores within the silica matrix. For H-(0.9) SO_3_-HS-30, the observed mesoporosity primarily originates from the interparticle voids formed during the aggregation of the silica nanoparticles as the water from the HS-30 suspension evaporated. The macropores within H-(0.9) SO_3_-0%-silica are derived from the removal of organic templates, whereas the mesopores originate from the aggregation of silica nanoparticles.

**Table 2:** The surface areas, acid density, and turnover frequency (TOF) of sulfonated silica monoliths

Catalyst	*S*_BET_ a (m^2^ g^−1^)	*S*_meso-BET_ (m^2^ g^−1^)	*V*_BJH_ b (cm^3^ g^−1^)	–SO_3_H density^c^ (mmol g^−1^)	TOF^d^ (h^−1^)
H-(0.9) SO_3_-TEOS monolith	9.4	9.0	0.007	0	—
H-(0.9) SO_3_-TEOS + SDS monolith	200.9	25.8	0.05	0.49	—
H-(0.9) SO_3_-HS-30	117.3	114.5	0.14	0.44	—
H-(0.9) SO_3_-0%-silica	243.2	241.6	0.67	0.71	10.9
H-(0.9) SO_3_-25%-silica	239.7	237.2	0.62	0.71	19.4
H-(0.9) SO_3_-50%-silica	225.3	220.2	0.57	0.67	24.1
H-(0.9) SO_3_-75%-silica	232.7	223.1	0.63	0.73	32.1
H-(2.7) SO_3_-75%-silica	258	252.7	0.68	0.69	27.8
H-75%-silica	219.7	212.1	0.61	0	—

a BET method applied to the N_2_ isotherm (*p*/*p*_0_ 0.05–0.3).

b Surface area and pore volume of mesopores determined by the BJH method.

c Based on the S content from elemental analysis.

d Based on the free fatty acid conversion in the first hour of the reaction.

For the emulsion-templated silica monoliths, all samples exhibited the presence of mesopores, with high mesopore volumes and BET surface areas. Indeed, the specific surface areas of these samples were mainly contributed by those mesopores (Table 2). As shown in Fig. 3a, all nitrogen adsorption–desorption isotherm profiles display pronounced hysteresis loops at high relative pressure, characteristic of type IV isotherms according to the IUPAC classification,^47^ confirming the presence of mesopores. The corresponding pore size distribution curves (Fig. 3b) further indicate that the majority of pores are in the range of 5 nm to 35 nm, consistent with the mesoporous structure.

**Fig. 3:**
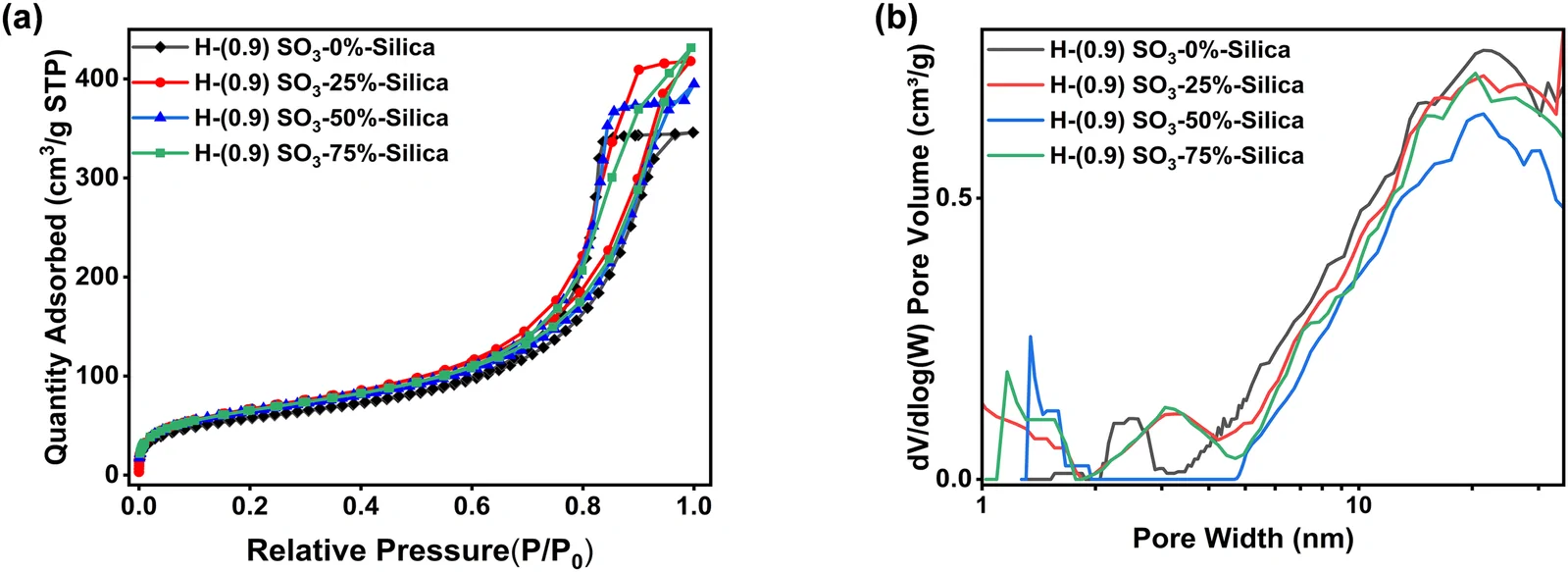
N_2_ sorption characterisation of emulsion-templated sulfonated silica monoliths. (a) N_2_ adsorption–desorption isotherm profiles. (b) Pore size distributions of the monoliths.

The mesoporosity is primarily attributed to the interparticle voids formed from aggregated silica nanoparticles. Notably, both the non-sulfonated and sulfonated emulsion-templated silica monoliths demonstrated nearly identical BET surface areas and mesopore volume, regardless of the emulsion ratio (Tables S1 and 2). This indicates that the variations in emulsion ratio only change the macroporosity but have minimal impact on the mesoporous structure or specific surface area of the silica monoliths.

Furthermore, the BET surface area showed a negligible change following sulfonation. As illustrated in Fig. S1(a and b), the isotherms and pore distribution remained largely unchanged before and after sulfonation. Even when the amount of 3-MPTMS was tripled in H-(2.7) SO_3_-75%-silica compared to H-(0.9) SO_3_-75%-silica, the surface area, mesopore volume and pore distribution only had a slight change (Table 2 and Fig. S1(c and d). These results confirm that the sulfonation process had limited influence on the mesoporosity and surface area, and the mesoporous structure was preserved well after sulfonation.

#### 3.1.5 XPS spectroscopy

XPS spectroscopy was employed to confirm the status of sulphur on the surface of modified silica monoliths. This was demonstrated using the 75% emulsion-templated silica monolith. The carbon (C 1s) XPS spectra (Fig. 4a) were the same for the monolith modified with 3-MPTMS and after oxidation to form –SO_3_H groups. Analysis of the S 2p region of these two monoliths confirmed the oxidation of thiol groups (–SH) into sulfonic acid groups (–SO_3_H) (Fig. 4b). The monoliths modified with 3-MPTMS (sample H-(0.9) SH-75%-silica) exhibited a characteristic doublet at 163.4 eV, corresponding to thiol species. Following H_2_O_2_ oxidation to convert –SH to –SO_3_H, the doublet peak is shifted to 168.4 eV, which is attributed to sulfonic acid functionalities, confirming complete conversion of thiol groups to sulfonic acid groups.^48^

**Fig. 4:**
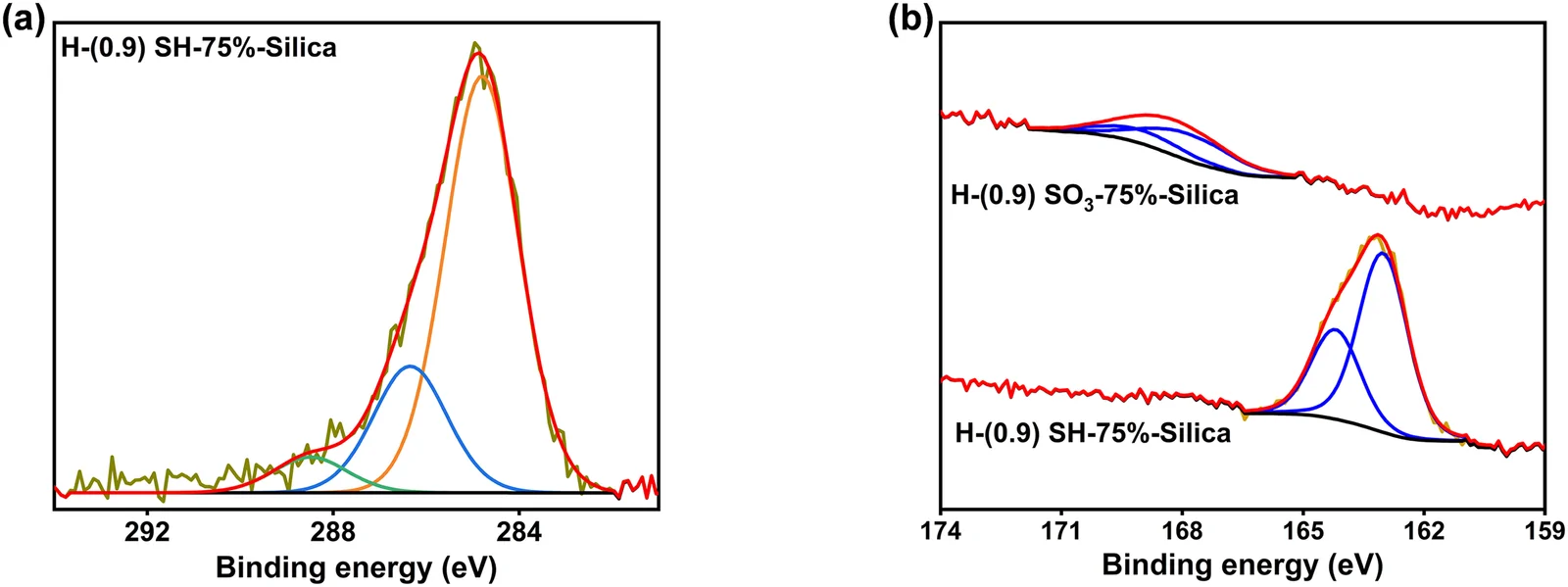
(a) XPS spectrum of C 1s for H-(0.9) SH-75%-silica. (b) XPS spectrum of S for H-(0.9) SH-75%-silica and H-(0.9) SO_3_-75%-silica.

#### 3.1.6 Thermal stability of sulfonated silica monoliths

The thermal stability of a catalyst is important for heterogeneous catalytic reactions at elevated temperatures. This can be evaluated by thermogravimetric analysis (TGA) on the sulfonated emulsion-templated monoliths. The TGA data (Fig. 5a) show that the non-sulfonated H-75% silica displays only a small mass loss around 100 °C, which is attributed to moisture desorption. For the emulsion-templated sulfonated monoliths, there are more significant mass losses up to around 100 °C. This is assigned to higher moisture content present in the silica monoliths with higher pore volumes. Importantly, the sulfonated silica monoliths exhibit an extra mass loss between ∼320 and 520 °C, assigned to the decomposition of propyl sulfonic groups.^49^ The size of this loss is about 5 wt% and is similar across the samples prepared with different emulsion ratios, indicating similar –SO_3_H loadings. No significant decomposition occurs below 320 °C, demonstrating that the –SO_3_H groups on silica are relatively thermally stable.

**Fig. 5:**
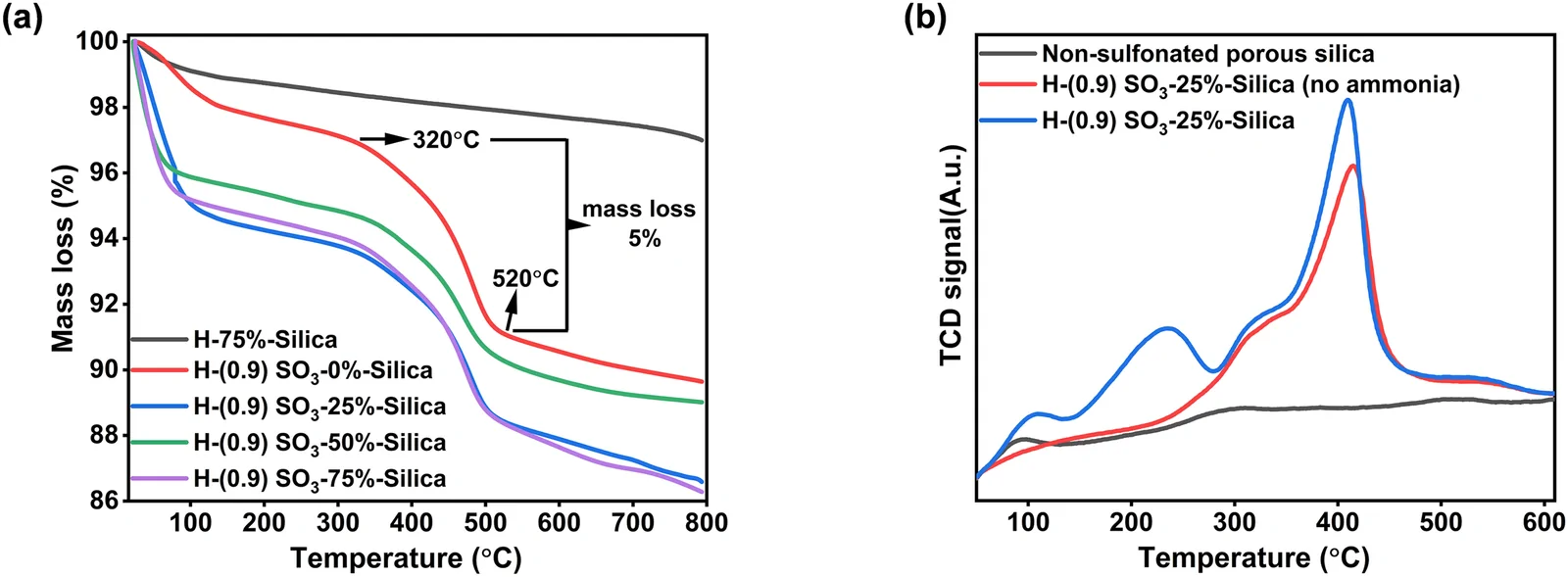
(a) TGA profiles for silica monoliths. (b) NH_3_-TPD profiles for non-sulfonated porous silica and H-(0.9) SO_3_-25%-silica without (blank sample) and with NH_3_ adsorption.

#### 3.1.7 Evaluation of –SO_3_H acidity

CHNS elemental analysis was conducted to evaluate the surface acidity of the sulfonated silica monolith based on the sulphur content. The density of –SO_3_H was calculated based on S % in the monoliths (Table S2) and is listed in Table 2. For the non-emulsion templated catalysts, H-(0.9) SO_3_-TEOS contained a negligible –SO_3_H content (below detection), whereas H-(0.9) SO_3_-TEOS + SDS and H-(0.9) SO_3_-HS-30 showed comparable acid-site densities of 0.49 and 0.44 mmol g^−1^, respectively. H-(0.9) SO_3_-0%-silica exhibited higher –SO_3_H density of 0.71 mmol g^−1^. The higher loadings of acidic sites are consistent with the larger surface areas and macroporosity of silica monoliths, which provide more accessible sites for grafting. For the emulsion-templated catalysts, all of them showed similarly high –SO_3_H density, in the range of 0.67–0.73 mmol g^−1^.

NH_3_-TPD analysis was conducted using NH_3_ as a probe molecule to characterise the acidic sites of the fabricated catalysts, with the desorption profiles indicative of the presence and relative strength of acidic sites. A thermal conductivity detector (TCD) was used in the NH_3_-TPD analysis. This means that the measurements could not differentiate between desorbed NH_3_ and the decomposition of propyl sulfonic groups. Fig. 5b shows the TCD signal profiles of a sulfonated sample with NH_3_ and in the absence of NH_3_. A non-sulfonated sample (H-75%-silica) was also measured as a control. It can be observed that the non-sulfonated sample exhibits only a minor desorption peak at low temperatures and no detectable signal at higher temperatures, suggesting the presence of limited surface acidity. In contrast, the sulfonated silica monolith displayed two desorption peaks below 300 °C and one strong peak in the high-temperature region (around 400 °C). However, the same sulfonated monoliths measured in the absence of NH_3_ shows no peaks below 250 °C but distinct peaks in the temperature range of 300–450 °C, although weaker than the sample measured in the presence of NH_3_. The TCD peak from the sample measured without NH_3_ is attributed to the decomposition of propyl sulfonic groups, as evidenced by the TGA data (Fig. 5a). Therefore, NH_3_-TPD was used only as qualitative evidence for the presence of acidic sites, and no quantitative acid density or acid strength could be derived from these measurements. The sulfonic acid site density was evaluated from the sulphur content determined by CHNS elemental analysis.

Based on the acid density evaluated from CHNS elemental analysis, the increased macroporosity in the silica monolith did not contribute to a higher acid density. A plausible explanation is that all materials offer similar numbers of surface silanol groups, yielding similar grafting contents. The presence of additional oxygen-containing sites, including phenolic –OH groups, as well as the silanol species generated through hydrolysis of the methoxy groups in grafted 3-MPTMS, contributes to the overall acidic sites.^50,51^

After HNO_3_ treatment of calcined monoliths, different procedures (washed and dried, washed but not dried, dried without washing, no drying & no washing) had been examined before grafting the silica with 3-MPTMS in H_2_O_2_ solution. It was found that the procedure after HNO_3_ treatment had a significant impact on –SO_3_H density (measured by CHNS elemental analysis and then calculated based on the S content) of the resulting sulfonated monoliths. Table 3 summarises the effect of residual HNO_3_ and water in the acid-treated monoliths on the –SO_3_H density of H-(0.9) SO_3_-75%-silica. Silica monoliths without HNO_3_ treatment, or those subjected to HNO_3_ treatment followed by washing procedures, showed relatively low –SO_3_H density. In contrast, samples retaining residual HNO_3_ showed higher –SO_3_H density. This enhancement is likely due to the accelerated hydrolysis and condensation of 3-MPTMS in the presence of residual H^+^, which provided more 3-MPTMS grafted on the silica surface. However, this explanation should be regarded as a possible hypothesis rather than direct evidence of the hydrolysis/condensation mechanism. Notably, catalysts containing both residual HNO_3_ and water (that was the sample with no washing and no drying after HNO_3_ treatment) exhibited the highest –SO_3_H density, indicating that more 3-MPTMS could be grafted on the silica monoliths in the coexistence of H^+^ and a small amount of water. This procedure was thus adopted as the standard procedure for the preparation of sulfonated silica monoliths. Further studies, such as ^29^Si solid-state NMR, would be required to verify the condensation state and incorporation of 3-MPTMS in the silica framework.

**Table 3:** Influence of HNO_3_ and water content in the preparation of silica monoliths on the –SO_3_H group density

Catalyst	–SO_3_H density^a^ (mmol g^−1^)
(0.9) SO_3_-75%-silica	0.44
H-(0.9) SO_3_-75%-silica (washed)	0.46
H-(0.9) SO_3_-75%-silica (0.1 M HNO_3_ dried in air)	0.56
H-(0.9) SO_3_-75%-silica (0.5 M HNO_3_ dried in air)	0.59
H-(0.9) SO_3_-75%-silica (1 M HNO_3_ dried in air)	0.53
H-(0.9) SO_3_-75%-silica (0.1 M HNO_3_ with water)	0.73

a Based on the S content measured by elemental analysis.

### 3.2 Catalytic performance

#### 3.2.1 Esterification of oleic acid-rich free fatty acid

The sulfonated silica materials prepared with and without emulsion templating were evaluated as heterogeneous catalysts for esterification, resulting in the formation of methyl oleate (Fig. S3). After the reaction, the resulting mixture was subjected to rotary evaporation at 50 °C for 6 h to remove residual methanol and by-product water. This improved the accuracy in determining the acid value of the product. There was concern if the esterification reaction would be continued during the evaporation process. It was envisaged that this was unlikely because methanol was removed very rapidly by rotary evaporation. A control experiment was further performed to compare the rotary evaporation at room temperature for 20 min (removing methanol) and at 50 °C for 6 h. This led to a similar conversion of fatty acid (44.19% *vs.* 45.16%).

For the non-emulsion-templated silica without sulfonate modification, the free fatty acid conversion was low (Fig. 6a), ranging from 5.8% to 6.6%, comparable to the conversion observed without a catalyst (6.8%). This behaviour is attributed to the absence or very low number of acidic sites (Si–OH) on the silica. The non-emulsion-templated sulfonated silica monoliths generally exhibited improved catalytic performance, except for the H-(0.9) SO_3_-TEOS monolith (7.2%). This poor performance associated with the H-(0.9) SO_3_-TEOS monolith could be attributed to its very low specific surface area, which restricted the 3-MPTMS grafting and thus resulting in low acid density. In contrast, the H-(0.9) SO_3_-TEOS + SDS monolith, H-(0.9) SO_3_-HS-30 and H-(0.9) SO_3_-0%-silica demonstrated higher catalytic performance, with free fatty acid conversions reaching 43.3%, 35.1%, and 66.6%, respectively. Although no emulsion templating was used in their preparation, these silica materials exhibited higher surface area and porosity (Table 2). This facilitated molecular diffusion during the reaction and provided a greater surface area for the grafting of 3-MPTMS. These structural advantages translated into improved catalytic performance for the esterification reaction.

**Fig. 6:**
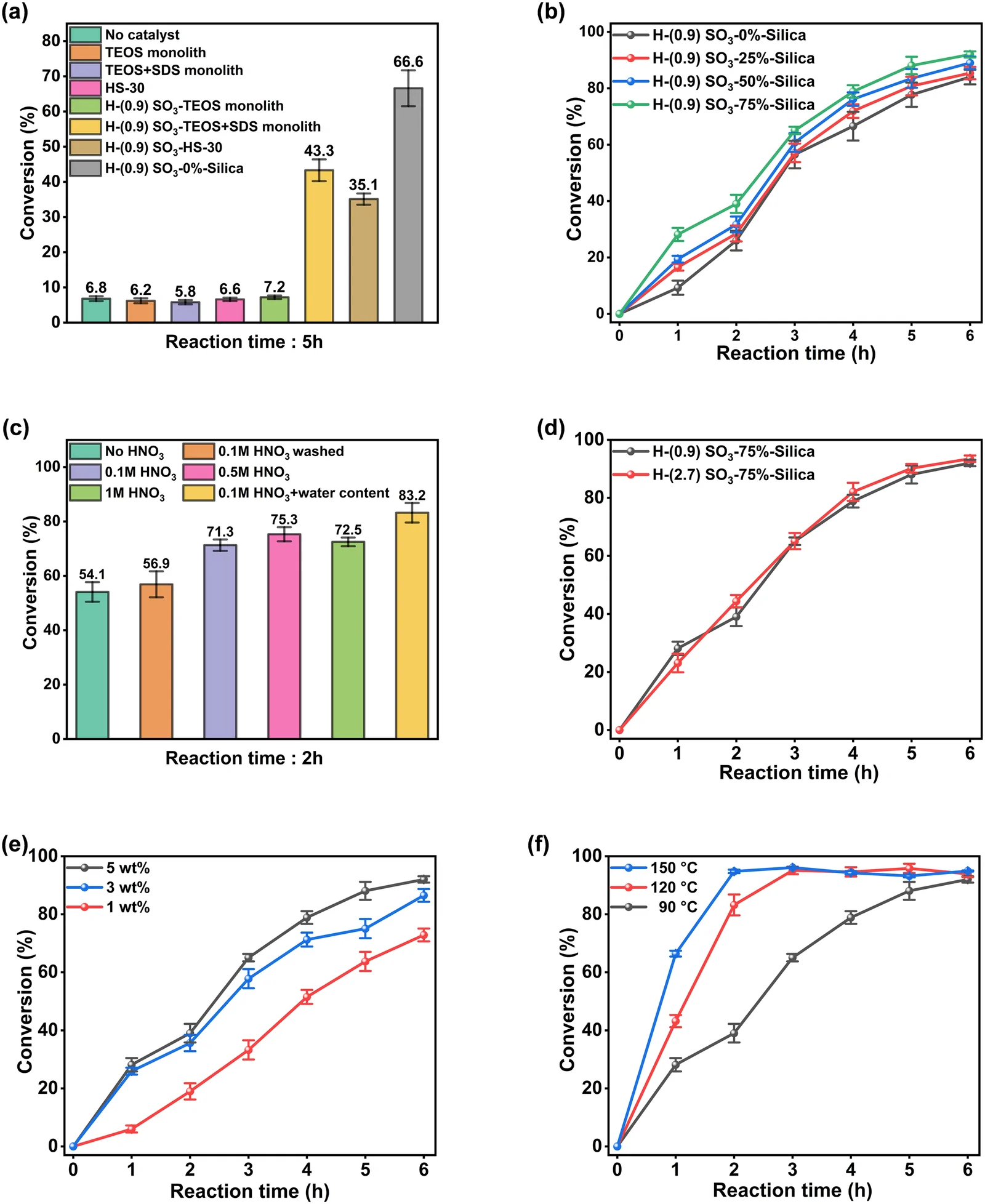
The conversion of free fatty acid in the esterification reaction with methanol under different reaction conditions using sulfonated silica monoliths as solid acid catalysts. (a) Non-emulsion templated silica materials as the catalysts. (b) The effect of emulsion ratio for emulsion-templated silica catalysts. (c) The effect of HNO_3_ treatment during the preparation of H-(0.9) SO_3_-75%-silica monoliths. (d) The effect of 3-MPTMS concentration during catalyst preparation. (e) The effect of catalyst loading for the catalyst H-(0.9) SO_3_-75%-silica. (f) The effect of reaction temperature for the catalyst H-(0.9) SO_3_-75%-silica. Standard reaction conditions: temperature 90 °C, catalyst loading: 5 wt% (relative to the oleic acid-rich free fatty acid feedstock mass), ratio of methanol to oleic acid: 10 : 1*.*

The introduction of emulsion-templated macropores in the silica materials was found to improve the reaction conversion further. As shown in Fig. 6b, all emulsion-templated catalysts exhibited significantly higher catalytic activity compared to their non-emulsion-templated counterparts. This enhancement is attributed to their higher acid site densities, along with the presence of macropores, which effectively promote molecular diffusion. Although the emulsion-templated catalysts possessed comparable acid densities and mesoporosity, the catalytic efficiency increased with higher emulsion ratios (Fig. 6b). This improvement is attributed to the formation of more macropores at higher emulsion ratios, which further facilitated mass transport during the esterification process. The turnover frequency (TOF) of the emulsion-templated catalysts (Table 2) exhibited the same trend, with higher TOF observed for the sulfonated silica monoliths prepared at higher emulsion ratios. Specifically, the TOF of H-(0.9) SO_3_-75%-silica reached 32.1 h^−1^, whereas H-(0.9) SO_3_-0%-silica showed only 9.3 h^−1^, confirming the significant role of macropores in enhancing mass transfer.

The influence of residual HNO_3_ and water in H-(0.9)-75%-silica on the grafting of 3-MPTMS to this monolith was investigated on the catalytic efficiency as the sulfonated catalyst H-(0.9) SO_3_-75%-silica. As shown in Fig. 6c, silica monoliths without HNO_3_ treatment, as well as those treated with HNO_3_ followed by washing, exhibited relatively low catalytic efficiency. The conversion of free fatty acid was around 55% after 2 hours of reaction at 120 °C. In contrast, catalysts treated with HNO_3_ and subsequently dried in air without washing displayed enhanced performance, achieving ∼72% free fatty acid conversion under the same reaction conditions. Further increasing HNO_3_ concentration did not improve catalytic efficiency, as no significant change in the free fatty acid conversions was observed. The catalyst prepared from the silica monolith containing both residual HNO_3_ and trace water (that is, no washing & no drying after the HNO_3_ treatment) exhibited the highest catalytic efficiency (83.2%). Overall, the catalytic performance follows the same trend as the density of acid sites (Table 3).

A higher concentration of 3-MPTMS was also used in the preparation of sample H-(2.7) SO_3_-75%-silica to investigate its effect on the catalytic performance. As shown in Fig. 6d, the increase of 3-MPTMS concentration did not lead to improved catalytic performance. According to Table 2, the acid density and mesoporosity of H-(2.7) SO_3_-75%-silica were of similar values compared to H-(0.9) SO_3_-75%-silica, suggesting that additional 3-MPTMS did not further contribute to the surface functionalization. This result indicates that the grafting of 3-MPTMS on the surface of fabricated silica monoliths reaches the saturation point at the loading of 0.9 g 3-MPTMS per gram of silica monolith, beyond which further increases in 3-MPTMS concentration do not lead to enhanced acid density and catalytic performance.

The effects of catalyst loading and reaction temperature on this esterification reaction were investigated. As illustrated in Fig. 6e, increasing catalyst loading results in a higher conversion of free fatty acids, which can be attributed to the increased number of available acidic sites. Fig. 6f presents the influence of reaction temperature on this esterification reaction. The esterification rate increases significantly with higher temperatures, reaching almost complete conversion (around 95%) within 2 hours at 150 °C. The accelerated reaction rate at elevated temperatures is expected, resulting from the enhanced molecular motion and collision between reactant molecules. Table S3 presents a comparison of various catalysts reported in the literature for the esterification reaction. This comparison should be interpreted as a qualitative benchmark rather than a direct performance ranking, because the reaction conditions vary among different studies. Under the tested conditions, the sulfonated hierarchical silica monoliths showed promising catalytic performance, achieving high total free fatty acid conversion within a relatively short reaction time, while their monolithic form enabled facile recovery and reuse.

#### 3.2.2 Acetalization of glycerol

The catalytic performance of the sulfonated silica monoliths was also evaluated by the acetalization of glycerol with acetone. As shown in Fig. 7a, using H-(0.9) SO_3_-75%-silica as the catalyst, the conversion of glycerol reached 98.6% within 30 min, while the selectivity toward solketal achieved 98% (Fig. 7b). The near-complete glycerol conversion should also be considered in the context of the large excess of acetone used in this reaction, which shifts the equilibrium toward solketal formation. The high conversion arises from the combined effects of catalytic activity and favourable thermodynamic driving force. As summarized in Table 4, H-(0.9) SO_3_-75%-silica exhibited a high TOF of 1155 h^−1^, together with a glycerol-based carbon balance of 99.4%. This high carbon recovery indicates that the carbon from glycerol was efficiently converted into the desired products, with negligible contribution from coke formation. The influence of macroporosity on catalytic performance is noted, exhibiting a similar trend to that observed in the esterification of oleic acid-rich free fatty acid, with catalysts prepared with higher emulsion ratios showing higher catalytic efficiency (Fig. 7a). H-(0.9) SO_3_-75%-silica exhibited consistently higher activity than those silica monoliths with lower emulsion ratios, reaching the equilibrium more rapidly. Since these catalysts possessed comparable acid densities and mesoporous structures, the enhanced activity can be primarily attributed to improved macroporosity, which facilitates mass transport. The solketal selectivity (Fig. 7b) was very similar across all catalysts, reaching 98%. The five-membered solketal was formed with much higher selectivity than the six-membered acetal because it is the more energetically favourable product. In contrast, the six-membered ring places one methyl group in an axial position in its chair conformation, which introduces steric repulsion with axial ring hydrogens and makes this isomer significantly less stable.^52^

**Fig. 7:**
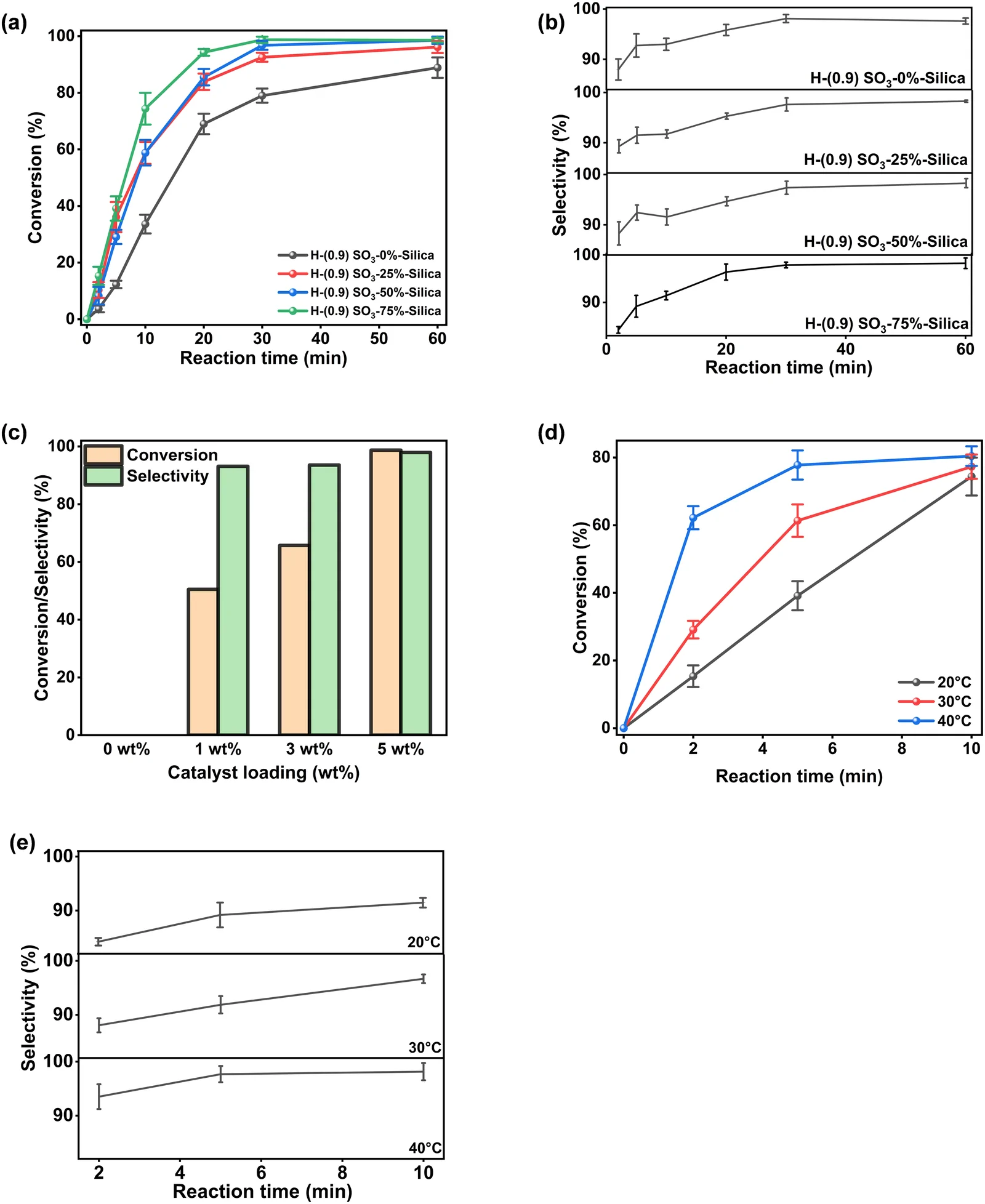
The conversion of glycerol and selectivity of solketal in the acetalization reaction with acetone under different reaction conditions with emulsion-templated sulfonated silica monoliths. (a) Effect of emulsion ratio on catalyst performance in glycerol conversion. (b) Effect of emulsion ratio on catalyst performance in solketal selectivity. (c) The effect of catalyst loading for the catalyst H-(0.9) SO_3_-75%-silica. (d) The effect of reaction temperature on glycerol conversion for the catalyst H-(0.9) SO_3_-75%-silica. (e) The effect of reaction temperature on solketal selectivity for the catalyst H-(0.9) SO_3_-75%-silica. Standard reaction conditions: temperature 20 °C, catalyst loading: 5 wt% (relative to the glycerol mass), ratio of acetone to glycerol: 10.5 : 1.

**Table 4:** Glycerol-based carbon balance and TOF of H-(0.9) SO_3_-75%-silica for the acetalization reaction

Catalyst	Initial glycerol (mmol)	Final solketal^a^ (mmol)	Final acetal (mmol)	Final glycerol (mmol)	Glycerol-based carbon balance (%)	TOF^b^ (h^−1^)
H-(0.9) SO_3_-75%-silica	13.6	13.06	0.267	0.19	99.4	1155

a Based on the glycerol conversion in 30 min.

b Based on the glycerol conversion during the initial 2 min of the reaction.

The effect of catalyst loading on reaction performance was investigated (Fig. 7c). In the absence of catalyst, the conversion of glycerol was negligible. When the 5 wt% catalyst was used, the glycerol conversion and solketal selectivity reached 81% and 98%, respectively, after 1 h at 20 °C. In comparison, lower catalyst loadings of 1 wt% and 3 wt% resulted in glycerol conversions of 40% and 52%, respectively, with the solketal selectivity remaining high at 94%.

Temperature is another important factor influencing the acetalization reaction, and therefore several reaction temperatures were evaluated. As shown in Fig. 7d, the reaction rate increases significantly with increasing temperature. At 40 °C, the glycerol conversion reached 62.2% within 2 min, compared to 29.0% at 30 °C and only 15.3% at 20 °C. As the reaction progressed toward equilibrium, the conversion values across different temperatures became closer, yielding 74.4%, 77.3%, and 80.4% at 20 °C, 30 °C, and 40 °C within 10 min, respectively. Fig. 7e shows the solketal selectivity at different reaction temperatures. A similar trend to glycerol conversion was observed: at 40 °C, the selectivity reached 93.5% within 2 min, compared to 88.1% and 84.2% at 30 °C and 20 °C, respectively. A higher proportion of the six-membered acetal is observed at short reaction times, while prolonged reaction leads to an increasing fraction of solketal, consistent with its greater thermodynamic stability.^53^ At 40 °C, the selectivity further increased to 98.1% after 10 min. Upon reaching equilibrium, a solketal selectivity of approximately 98% was achieved at both 20 °C and 40 °C. These results demonstrate that higher reaction temperatures not only shorten the time required to achieve high selectivity but also enhance the overall reaction rate, confirming that elevating the temperature is beneficial for the formation of solketal.

Table S4 presents a comparison of various catalysts reported for the acetalization of glycerol. Similarly, this comparison is intended to provide a qualitative benchmark because the reaction conditions differ among the reported studies. Under the tested conditions in this work, H-(0.9) SO_3_-75%-silica showed promising catalytic performance, achieving 98.6% glycerol conversion within 0.5 h at 20 °C with 98% solketal selectivity. This indicates that the catalyst is highly effective for glycerol acetalization under mild conditions.

### 3.3 Structure–performance relationship and catalytic pathway

The catalytic results indicate that the activity of the sulfonated silica monoliths is not determined by sulfonic acid density alone, but by how effectively these acid sites can be accessed and utilised during liquid-phase reactions. CHNS elemental analysis showed that the emulsion-templated catalysts possessed comparable –SO_3_H densities, while N_2_ sorption results showed that they also had similar mesoporous structures and that the mesoporous structure was largely retained after sulfonic acid functionalisation. Therefore, the improved catalytic performance observed for the highly emulsion-templated monoliths is more closely associated with enhanced macropore connectivity and acid site accessibility. The retained BET surface area and mesopore volume after functionalisation also suggest that 3-MPTMS grafting did not severely block the mesoporous structure. Thus, the accessible sulfonic acid sites are likely located on open silica surfaces, including macropore walls and external pore surfaces, where reactants can reach them more readily. The interconnected macropores provide liquid transport pathways, allowing fatty acids, methanol, glycerol, acetone and reaction products to diffuse through the monolithic catalyst more efficiently.

The catalytic behaviour is consistent with typical Brønsted acid-catalysed pathways. In the esterification of the oleic acid-rich fatty acid feedstock, surface –SO_3_H groups protonate the carbonyl oxygen of free fatty acids, increasing the electrophilicity of the carbonyl carbon and promoting nucleophilic attack by methanol, followed by ester formation and water release.^54^ In glycerol acetalization, the sulfonic acid sites activate acetone through carbonyl protonation, enabling nucleophilic attack by glycerol, followed by dehydration and cyclisation to form solketal.^55^ Therefore, the high catalytic performance of the emulsion-templated sulfonated silica monoliths arises from the combination of accessible Brønsted acid sites and interconnected macro–mesoporous transport pathways, rather than from acid density alone.

### 3.4 Catalyst reusability

To assess the reusability of the fabricated catalysts, H-(0.9) SO_3_-50%-silica was used as an example and was subjected to five consecutive catalytic cycles in both esterification and acetalization reactions. In the esterification of oleic acid-rich free fatty acid, the conversion decreased slightly from 94.9% to 83.3% by the third cycle (Fig. 8a) and then remained stable thereafter. A similar trend was observed for the acetalization of glycerol (Fig. 8b), where the conversion decreased from 76% to 60% after the second cycle and stabilized in subsequent runs. The solketal selectivity remained constant at approximately 98% throughout all five cycles.

**Fig. 8:**
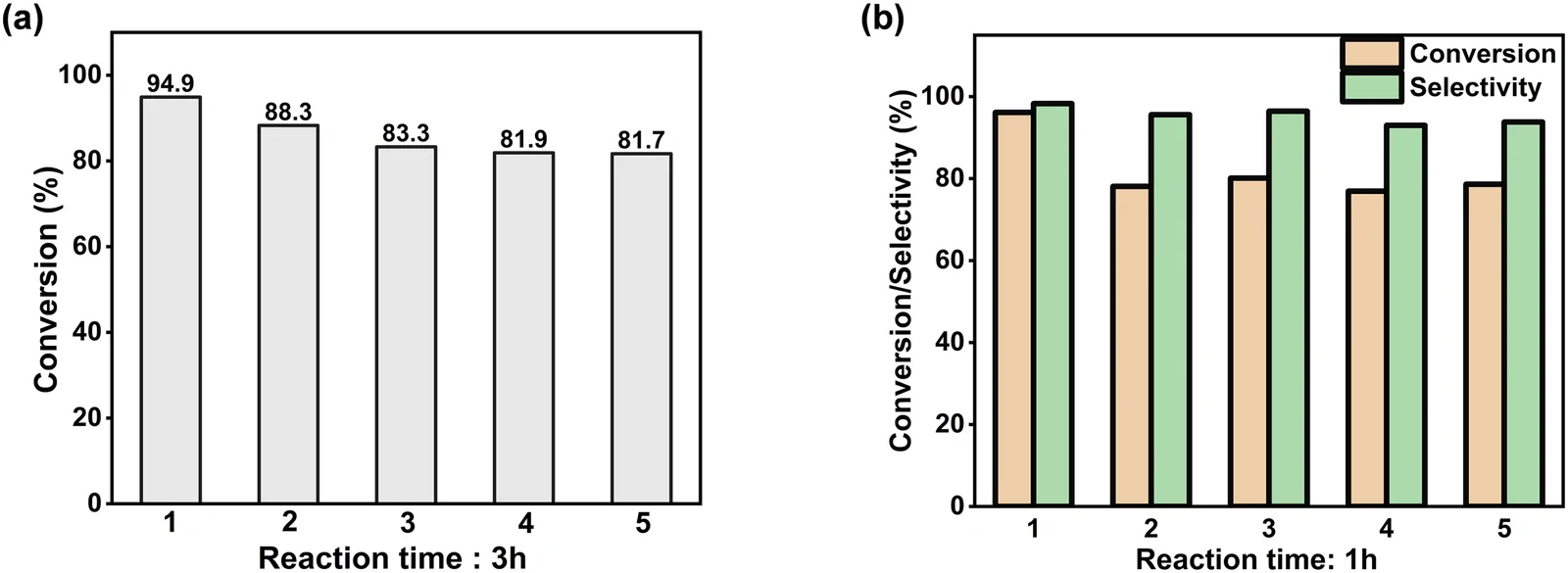
Evaluation of catalyst reusability and properties of the spent catalyst, using the catalyst H-(0.9) SO_3_-50%-silica as an example. (a) The free fatty acid conversion in 5 consecutive cycles using H-(0.9) SO_3_-50%-silica. Reaction conditions: temperature 120 °C, catalyst loading: 5 wt% (relative to the oleic acid-rich free fatty acid feedstock mass), ratio of methanol to oleic acid: 10 : 1. (b) The glycerol conversion and solketal selectivity in 5 consecutive cycles using H-(0.9) SO_3_-50%-silica. Reaction conditions: temperature 20 °C, catalyst loading: 5 wt% (relative to the glycerol mass), ratio of acetone to glycerol: 10.5 : 1.

The spent catalyst was characterized by various techniques, with attempts to gain insight into the reason for the slight decrease of catalytic efficiency. Fig. S4a and b show similar adsorption–desorption isotherm profiles and pore size distributions for the fresh and spent catalysts. The specific surface area and acid density of the spent catalyst decreased slightly, compared with the fresh catalyst (Table S5). The acid density was determined by direct titration with KOH solution (0.1 M). The acid density of the fresh catalyst H-(0.9) SO_3_-50%-silica was 0.59 mmol g^−1^, lower than the value (0.67 mmol g^−1^) calculated from the S content determined from elemental analysis (Tables S5 and 2). This confirms that not all S is accessible as –SO_3_H. It should also be mentioned that the acid density determined by titration also includes the contribution from the Si–OH groups. The FT-IR results indicate that the framework of spent catalysts was retained, as evidenced by the Si–O–Si vibration bands at ∼800 and ∼1050 cm^−1^. The C–H stretching band at 2850 cm^−1^ is detectable after five catalytic cycles in both reactions (Fig. S4c). The TGA results showed a slightly increased mass loss in the range of 320–520 °C after five reaction cycles in both reactions (Fig. S4d). This suggests that no substantial decomposition or loss of propyl sulfonic acid groups occurred during reuse, while the slight increase in mass loss may be attributed to organic deposits or strongly adsorbed reactants/products remaining on the catalyst surface.

CHNS elemental analysis was also performed on the fresh and the spent catalyst after five reaction cycles to evaluate changes in carbon and sulphur contents. After each cycle, the used catalyst was washed three times by soaking in ethyl acetate for esterification or ethanol for acetalization for 1 h per wash to remove residual reactants and products. As shown in Table S6, the carbon content increased from 2.24% in the fresh catalyst to 2.82% and 2.96% in the spent catalyst after esterification and acetalization, respectively, which may be attributed to minor organic deposits or strongly adsorbed reactants/products remaining on the catalyst surface during reuse. This is consistent with the decreased BET surface area observed for the spent catalyst, suggesting partial surface or pore blockage by deposited organic species. In contrast, the sulphur content showed a slight decrease from 2.15% to 2.02% and 1.93%. However, this does not necessarily indicate the loss of sulfonic groups due to the increased C content.

In order to directly confirm the possible leaching of –SO_3_H from the catalyst, an inductively coupled plasma (ICP) analysis of S content was performed on the liquid phase of the esterification reaction of oleic acid and methanol with H-(0.9) SO3-75%-silica as the catalyst after 5 h reaction at 90 °C and removal of the catalyst by filtration. The ICP result indicated a loss of 5.6% of the initial acid sites. This largely aligns with the loss of FFA conversion between the first and second reactions. All these results suggest that the catalyst exhibited good thermal stability and the slight decrease in FFA and glycerol conversion after reuse was mainly associated with the slight leaching of –SO_3_H groups and minor organic deposition or strongly adsorbed reactants/products.

A hot filtration test was conducted to evaluate the leaching stability of the catalyst in the esterification reaction. The esterification reaction was first carried out in an autoclave at 90 °C for 2 h using H-(0.9) SO_3_-50%-silica as the catalyst. For safety reasons, the autoclave could not be opened directly at 90 °C because of the internal pressure. Therefore, the reactor was allowed to cool for 15 min until the temperature decreased to around 50 °C. The liquid reaction mixture was then transferred into another autoclave, while the monolithic catalyst was removed from the system. The catalyst-free liquid phase was subsequently reacted for another 2 h at 90 °C under the same conditions.

The free fatty acid conversion reached 33.2% after the initial 2 h reaction in the presence of H-(0.9) SO_3_-50%-silica and increased only slightly to 35.3% after the additional 2 h reaction without the solid catalyst. In contrast, the free fatty acid conversion reached 76.2% after 4 h when the catalyst remained in the reaction system. This clear difference indicates that further esterification after catalyst removal was very limited, suggesting negligible leaching of catalytically active acid species into the liquid phase. The minor increase can be attributed to the low level of background esterification promoted by the intrinsic acidity of the fatty acid feedstock, as the blank reaction without catalyst showed 6.8% free fatty acid conversion at 90 °C after 5 h (Fig. 6a).

## 4. Conclusion

In summary, we fabricated sulfonated hierarchical silica monolith catalysts *via* an emulsion-templating route that are highly active, readily recyclable, and easy to recover. Commercially available silica colloids (HS-30 dispersion) were used as the building blocks to produce the silica monoliths. This provided the silica materials with interconnected macroporosity, tuneable macroporosity by varying the emulsion ratios, and high specific surface area. These silica monoliths were grafted with 3-MPTMS, followed by oxidation with H_2_O_2_ under mild conditions. We observed that treating the silica monolith with HNO_3_ before grafting with 3-MPTMS resulting in a higher density of –SO_3_H. Particularly, no washing and no drying after HNO_3_ treatment, that is, directly using these acid-treated monoliths for grafting with 3-MPTMS, generated the sulfonated silica monoliths with the highest density of –SO_3_H, which translated to higher catalytic activity as a solid acid catalyst. This strategy integrates commercial colloidal silica, emulsion-templated hierarchical porosity and post-synthetic sulfonic acid functionalisation into a recoverable monolithic solid acid catalyst with accessible acid sites and improved mass-transport potential.

The catalysts exhibited high performance in both oleic acid-rich free fatty acid esterification and glycerol acetalization. In esterification, free fatty acid conversion reached 95% at 90 °C within 6 h. In acetalization, the solketal selectivity remained above 98% across all fabricated catalysts and a conversion of 98.6% was obtained within 30 min at 20 °C. Furthermore, these monolithic catalysts could be separated and recycled easily. The catalysts showed high reusability over five consecutive cycles with only a minor decrease in conversion. This was explained by the slightly deteriorated acidic density and surface area of the spent catalyst. Overall, sulfonated hierarchical silica monoliths have been fabricated and evaluated as an efficient, reusable solid acid catalyst, providing an effective and environmentally friendly method for producing biodiesel and solketal.

## Author contributions

B. Z.: methodology, data curation, original draft preparation, editing. P. Y.: N_2_ sorption study. D. R. I.: NH_3_-TPD measurement and discussion. A. G. & A. S.: Hg intrusion porosimetry measurement and discussion. H. Z.: conceptualization, supervision, funding acquisition, draft review, editing. All authors have read and agreed to this version of the manuscript.

## Conflicts of interest

The authors declare no conflicts of interest.

## Supplementary Material

CY-016-D6CY00824K-s001

## Data Availability

Data underpinning the findings of this study can be accessed by contacting the corresponding author upon making a reasonable request. Supplementary information (SI) is available. See DOI: https://doi.org/10.1039/d6cy00824k.
