# Supplementary material for: Emulsion-templated hierarchical silica monoliths functionalized with sulfonic acid for esterification and acetalization reactions
Source: Catal Sci Technol. 2026 Sep 21;16(22):7719–36. doi: 10.1039/d6cy00824k (PMC13636408; doi:10.1039/d6cy00824k)
Supplement: Supplementary file 2 [file CY-016-D6CY00824K-s001.pdf]

Supplementary Information (SI) for Catalysis Science & Technology.
This journal is © The Royal Society of Chemistry 2026

Supporting Information

Emulsion-Templated Hierarchical Silica Monoliths Functionalized with Sulfonic Acid for Esterification and Acetalization Reactions

Binhang Zhao [a], Pan Yang [a], Donald R Inns [a], Angela Gondolini [b], Alessandra Sanson [b], Haifei Zhang [a*]

[a] Department of Chemistry, University of Liverpool, Crown Street, Liverpool, L69 7ZD, UK.

[b] National Research Council of Italy, Institute of Science, Technology and Sustainability for Ceramics (CNR-ISSMC), Via Granarolo 64, 48018, Faenza, Italy.

[*] Corresponding author: zhanghf@liverpool.ac.uk.

Supporting information content:

Characterization methods: pages S2

Figure S1-S4: pages S3-S6

Table S1-S6: pages S7-S12

References: pages S13

Characterization methods

X-ray photoelectron spectroscopy (XPS): For XPS measurements, survey spectra were collected with a pass energy of 160 eV, while high-resolution spectra were acquired using a pass energy of 20 eV, a step size of 0.1 eV, and a sweep time of 60 s. Charge neutralisation was achieved using an electron flood gun (filament current 0.38 A, charge balance 2 V, filament bias 4.2 V). All measurements were performed under ultra-high vacuum conditions, with a base pressure below 9×10^{-9} Torr, at room temperature (294 K). Data processing and analysis were conducted using CasaXPS (v2.3.25PR1.0). Binding energies were referenced to adventitious carbon at 284.8 eV. Peak fitting was performed using a Shirley background, and Voigt-like line shapes (LA(1.53, 243)) were applied to the S 2p, C 1s, Si 2p, and O 1s regions. The S 2p spectra were fitted as a spin–orbit doublet with a fixed splitting of 1.16 eV.

Ammonia temperature-programmed desorption (NH_3-TPD) measurements: Each test used approximately 0.1 g of sample, with the actual mass precisely recorded for each sample. Before desorption, the samples were pretreated under a helium flow (50 mL min^{-1}) at 200 °C for one hour with a heating rate of 10 °C min^{-1}. After cooling, the samples were exposed to 5% NH_3/He (30 mL min^{-1}) at 50 °C for 30 minutes, followed by helium purging (30 mL min^{-1}) for 30 minutes to remove physiosorbed ammonia. Desorption was then carried out from 50 °C to 700 °C at a ramp rate of 10 °C min^{-1} and held at the final temperature for 1h. Calibration was achieved using repeated pulses of 5% NH_3/He via a 507 μL sample loop.

GC-MS analysis for esterification of oleic acid and methanol: The analyses were performed using an Agilent 7890B gas chromatograph fitted with a VF-WAXms capillary column (25 m × 250 μm × 0.20 μm) and coupled to an Agilent 5977A mass spectrometer operating in electron-impact (EI) ionization mode at 70 eV. Helium was used as the carrier gas at a constant flow rate of 1 mL min^{-1}. The injector temperature was set to 250 °C, and 1 μL of sample was injected with a split ratio of 50:1. The oven temperature was programmed from 30 °C to 250 °C at a rate of 15 °C min^{-1} and held at 250 °C for 10 min. Compound assignment was performed based on mass spectral analysis.

GC-MS analysis for the acetalization of glycerol with acetone: The GC-MS instrument, column, and operating parameters were identical to those described above, except for the oven temperature program. The oven temperature was ramped from 30 °C to 120 °C at a rate of 20 °C min^{-1} and held for 4 min. GC-MS was employed for compound identification, while quantification of solketal and acetal was carried out using GC-FID under the same chromatographic conditions. The FID detector temperature was maintained at 300 °C.

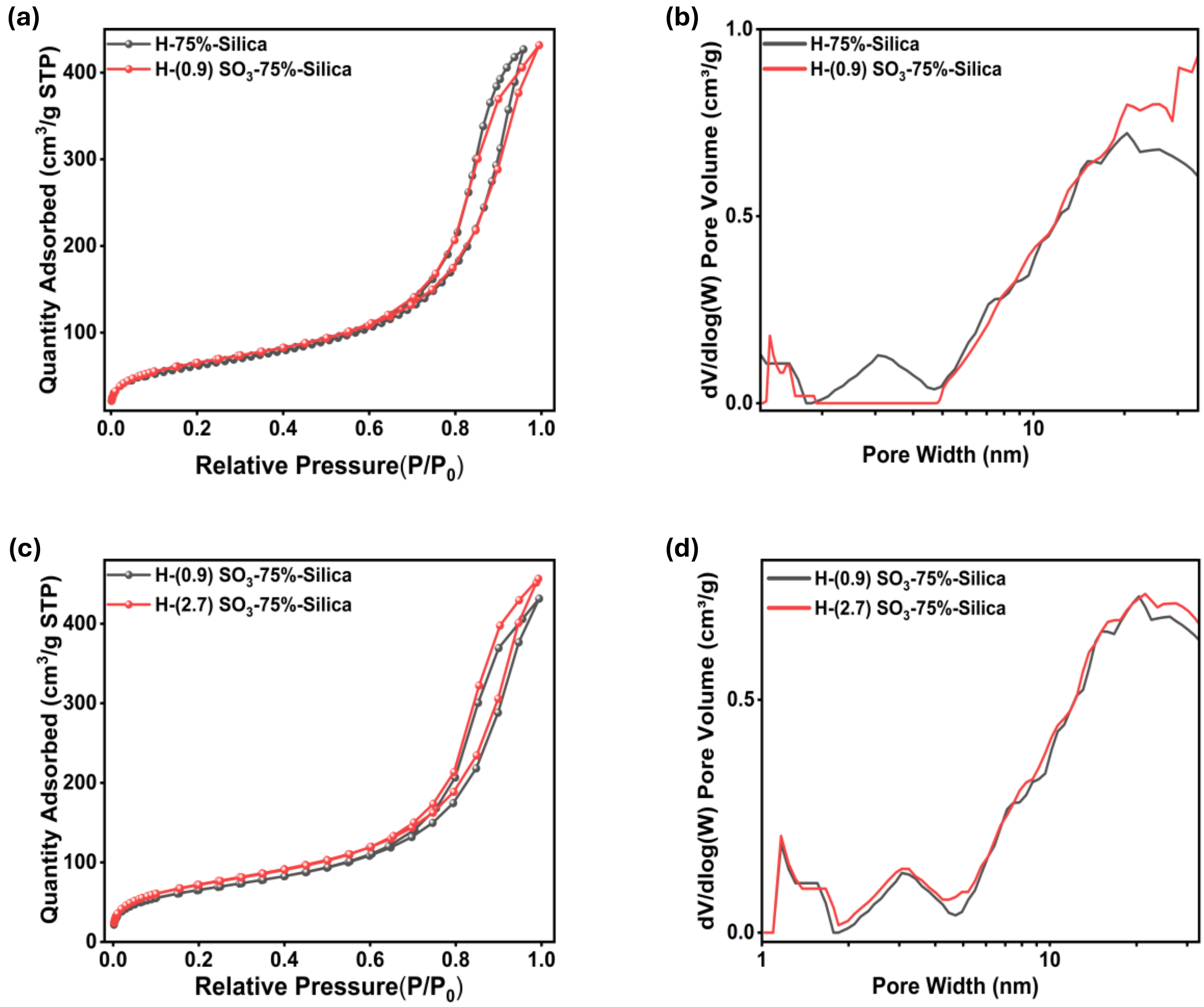

Fig. S1. Characterization of calcined silica and sulfonated silica monoliths. (a) N_2 adsorption-desorption isotherms of H-75%-Silica and H-(0.9) SO_3-75%-Silica. (b) Pore distribution of H-75%-Silica and H-(0.9) SO_3-75%-Silica. (c) N_2 adsorption-desorption isotherms of H-(0.9) SO_3-75%-Silica and H-(2.7) SO_3-75%-Silica. (d) Pore distribution of H-(0.9) SO_3-75%-Silica and H-(2.7) SO_3-75%-Silica.

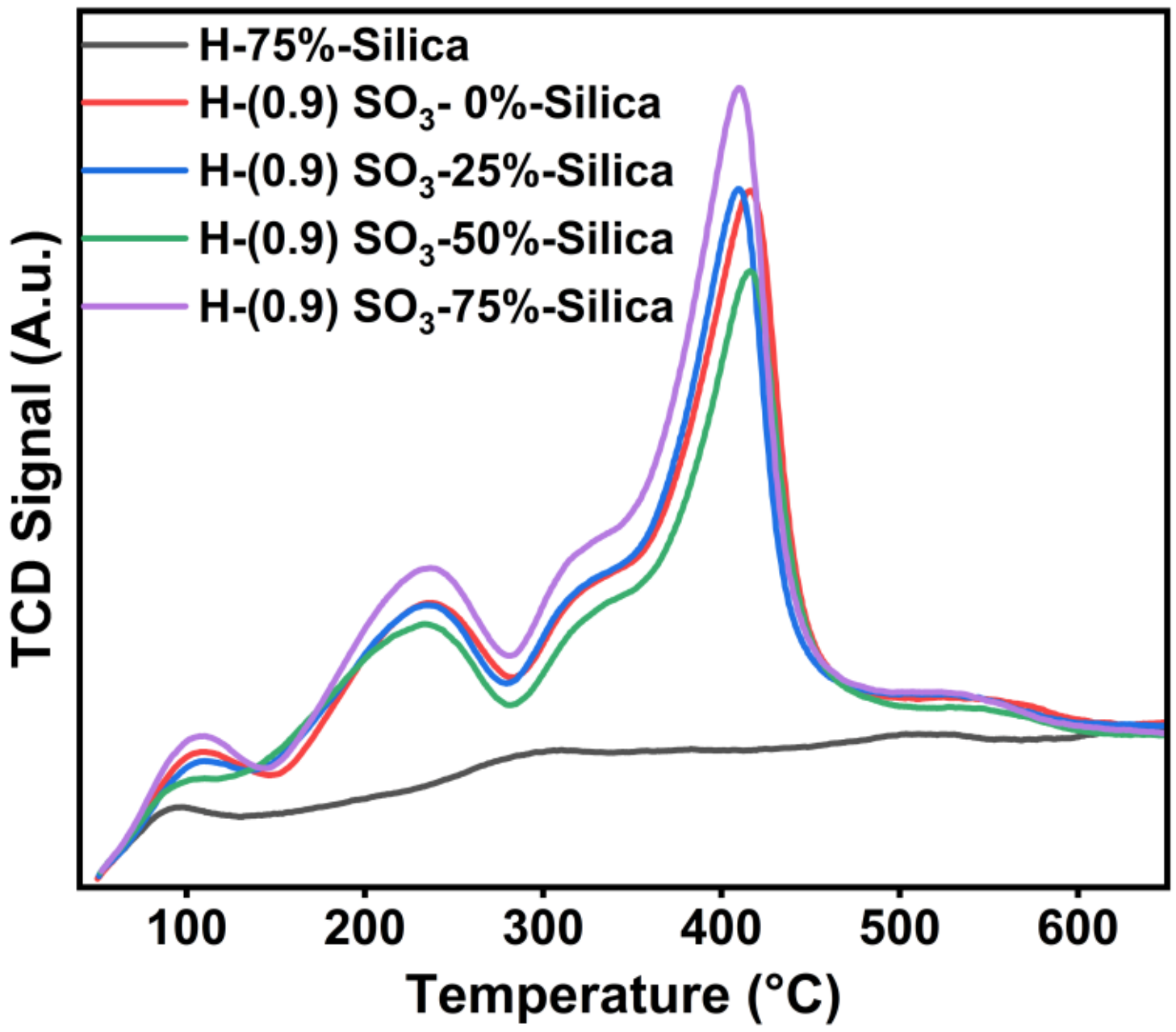

Fig. S2. NH_3-TPD profile of sulfonated silica monoliths, in comparison with the calcined silica monolith (No 3-MPTMS modification), measured with a thermal conductivity detector.

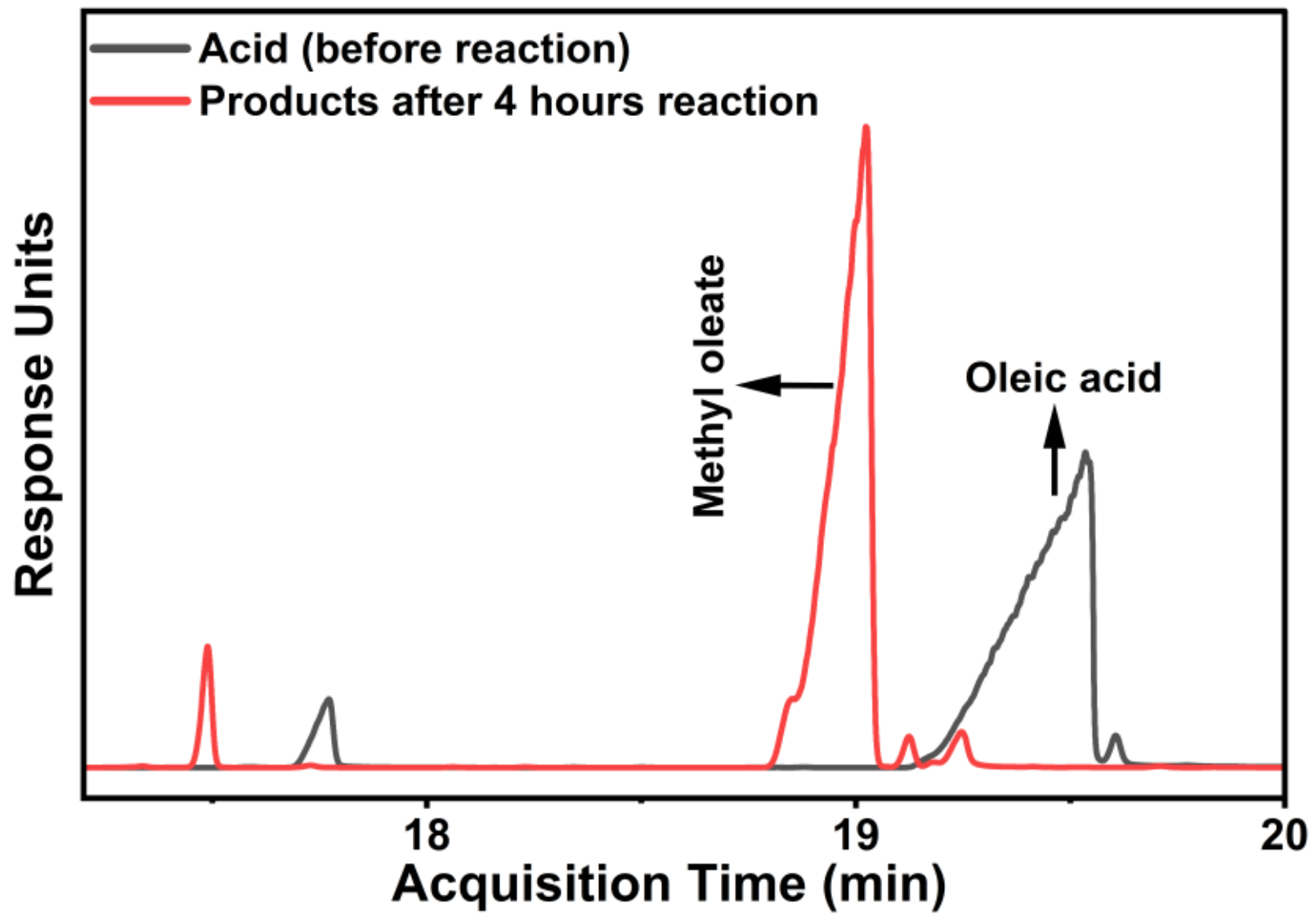

Fig. S3. GC–FID chromatograms before and after reaction over H-(0.9) SO_3-75%-Silica (120 °C, 2 h, catalyst loading 3 wt%). The oleic acid-rich free fatty acid feedstock (Sigma-Aldrich, 65–88% oleic acid) was converted to methyl oleate. Minor peaks arise from other fatty acids (black) and their corresponding methyl esters (red). For this analysis, the remaining product after evaporation by rotary evaporation was diluted with methanol at a volume ratio of 1:9 prior to analysis.

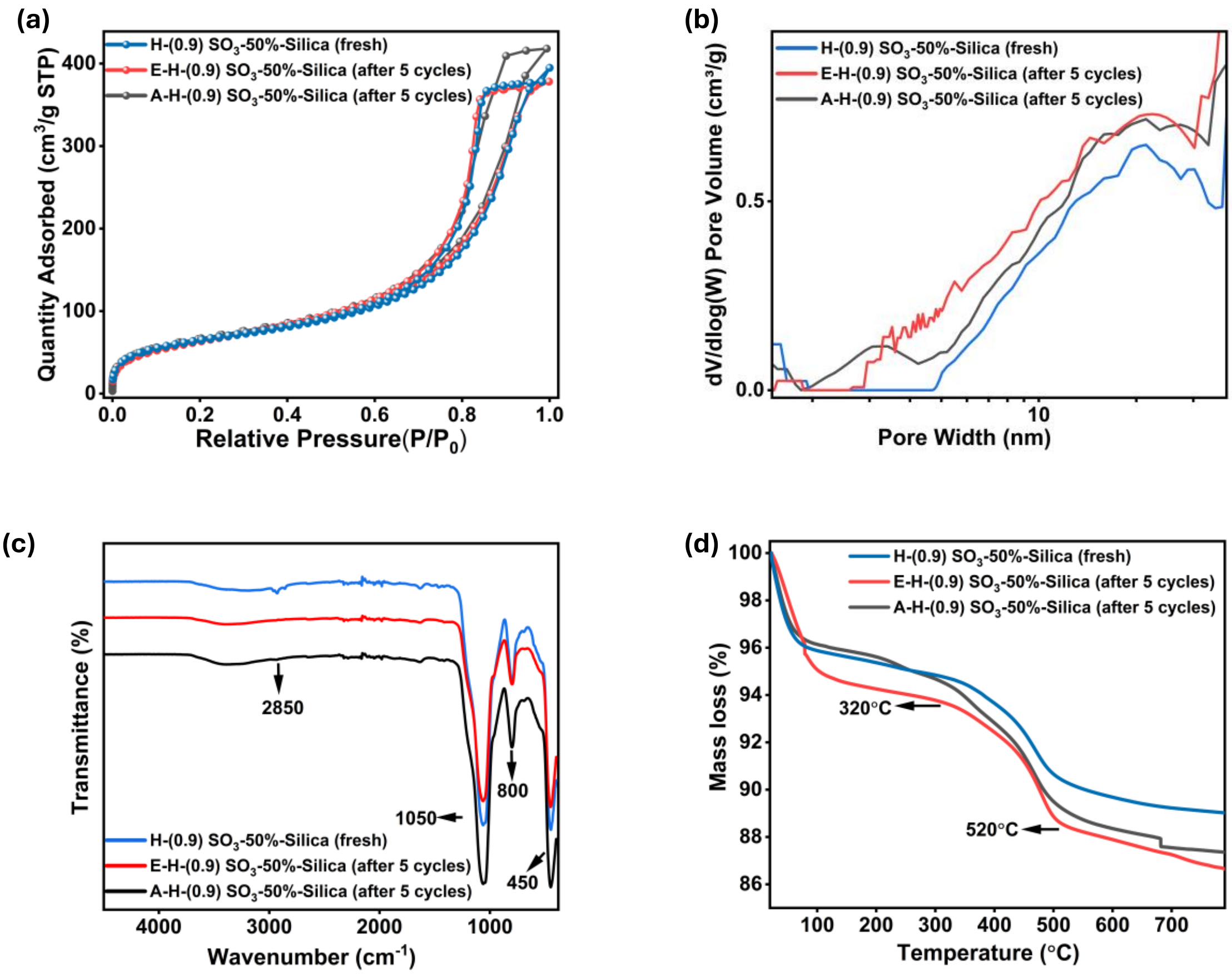

Fig. S4. Characterisation of spent catalysts after 5 cycles of reactions, compared with the fresh catalyst H-(0.9) SO_3-50%-Silica. (a) N_2 adsorption-desorption isothermal profiles. (b) Pore size distributions calculated from N_2 sorption data. (c) FT-IR spectra. (d) TGA profiles.

Table S1. BET surface area, mesopore surface area and BJH pore volume of non-sulfonated silica monoliths (after calcination and HNO_3 treatment, but no grafting of 3-MPTMs).

Catalyst	S_{BET} [a] (m^2/g)	$S_{meso\text{-}BET}$ (m^2/g)	V_{BJH} [b] (cm^3/g)
H-0%-Silica	236.5	231.3	0.68
H-25%-Silica	222.8	213.6	0.61
H-50%-Silica	224.9	211.5	0.58
H-75%-Silica	219.7	212.1	0.61

[a] BET method applied to the N_2 isothermal (p/p_0 0.05-0.3). [b] Surface area and pore volume of mesopores by BJH method.

Table S2. CHNS elementary analysis data for sulfonated samples.

Catalyst	C (%)	H (%)	S (%)
H-(0.9) SO_3-TEOS+SDS monolith	1.71	1.39	1.57
H-(0.9) SO_3-HS-30	1.52	0.73	1.41
H-(0.9) SO_3-0%-Silica	2.56	1.06	2.28
H-(0.9) SO_3-25%-Silica	2.54	1.01	2.27
H-(0.9) SO_3-50%-Silica	2.24	0.94	2.15
H-(0.9) SO_3-75%-Silica	2.68	1.12	2.34
H-(2.7) SO_3-75%-Silica	2.41	0.95	2.21

Table S3. A comparative analysis of catalytic activity in the esterification reaction of oleic acid with methanol.

Catalyst	Temperature (°C)	Catalyst loading [a] (wt%)	Molar ratio	Time (h)	Conversion (%)	Reference
xAIL@NH_2-UiO-66	75	5	1:14	6	96.8	[1]
$H_3PMo_{12}O_{40}$@EB-COF	70	7	1:8	8	95	[2]
H-(0.6) SO_3-0.5-Z	120	5	1:10	3	91	[3]
$TiFe_2O_4$@SiO_2-SO_3H	60	0.02g	1:4.5	3	98	[4]
Cation exchange resin	80	20	1:9	5	91.6	[5]
0.3HPW/OMS-SO_3H-5	120	1.12	1:30	10	90	[6]
Amberlyst-15	60	7	1:7	4	85	[7]
H-(0.9) SO_3-75%-Silica	120	5	1:10	2	83.2	Present work
H-(0.9) SO_3-75%-Silica	120	5	1:10	3	95.6	Present work

[a] catalyst loading based on the mass of oleic acid.

Table S4. A comparative analysis of catalytic activity in the acetalization of glycerol with acetone.

Catalyst	Temperature (°C)	Catalyst loading (wt%)^a	Molar ratio^b	Time (h)	Conversion (%)	Selectivity (%)	Reference
UAV-59	25	5	1:10	2	90	98	[8]
H-TiPOx	RT	4	1:4	8	84	99	[9]
UiO-SO_3H− 0.2	60	5	1:10	4	70.3	99.8	[10]
5 wt% Cu-FSM− 16	60	5	1:2	2.5	85	97.8	[11]
HPMo/AlTUD-1	60	10	1:6	3	78	94	[12]
rGo-SO_3H	RT	5	1:12	1	95	97	[13]
H_3PW_{12}@*Aptes*SBA-15	25	0.373 mmol H^+/g	1:15	1	97	97	[14]
RhMOP-SO_3H	RT	0.5	1:4	1	86	97	[15]
Amberlyst-15	60	1	1:4	3	68.75	-	[16]
H-(0.9) SO_3-75%-Silica	20	5	1:10.5	0.5	98.6	98	Present work

^{a}: The catalyst loading is based on the mass of glycerol; ^{b} the molar ratio of glycerol to acetone. RT: room temperature.

Table S5. Surface area and acid density of original and used catalyst. (after 5 cycles).

Catalyst	BET surface area (m^2/g) [a]	acid density (mmol/g) [b]
H-(0.9) SO_3-50%-Silica	220.2	0.59
E-H-(0.9) SO_3-50%-Silica (after 5 cycles) [c]	210.7	0.54
A-H-(0.9) SO_3-50%-Silica (after 5 cycles) [d]	213.2	0.56

[a]: BET method applied to the N_2 isothermal (p/p_0 0.05-0.3). [b]: Based on acid-base titration, the values only indicative of the small changes of acid density between fresh and spent catalysts. For the titration, samples (≥50 mg) were dispersed in 20 mL of a 1 mol/L NaCl solution and soaked for 24 h to promote the exchange of protons in the material with Na^+ ions. Phenolphthalein was used as the indicator, and the released protons were subsequently quantified by titration with a NaOH solution (5 mmol/L). [c]: E-H-(0.9) SO_3-50%-Silica (after 5 cycles) indicates the catalyst after 5 cycles in esterification. [d]: A-H-(0.9) SO_3-50%-Silica (after 5 cycles) indicates the catalyst after 5 cycles in acetalization.

Table S6. CHNS elementary analysis data for fresh and spent samples.

Catalyst	C (%)	H (%)	S (%)
H-(0.9) SO_3-50%-Silica	2.24	0.94	2.15
[a]E-H-(0.9) SO_3-50%-Silica	2.82	0.9	2.02
[b]A-H-(0.9) SO_3-50%-Silica	2.96	0.92	1.93

[a]: E-H-(0.9) SO_3-50%-Silica (after 5 cycles) indicates the catalyst after 5 cycles in esterification; [b]: A-H-(0.9) SO_3-50%-Silica (after 5 cycles) indicates the catalyst after 5 cycles in acetalization.

Reference

1. P. Lu, H. Li, M. Li, J. Chen, C. Ye, H. Wang and T. Qiu, *Fuel*, 2022, **324**, 124537.
2. Y. Zhao and G. Li, *J. Inorg. Organomet. Polym. Mater.*, 2025, **35**, 46–57.
3. B. Zhao, P. Yang, N. Zhang, D. R. Inns, E. F. Kozhevnikova, A. P. Katsoulidis, I. V. Kozhevnikov, A. Steiner and H. Zhang, *Chem. Commun.*, 2024, **60**, 13356–13359.
4. M. Y. Saleh, A. K. O. Aldulaimi, S. M. Saeed and A. H. Adhab, *Heliyon*, 2024, **10**, e26286.
5. J. Zhu, W. Jiang, Z. Yuan, J. Lu and J. Ding, *Process Saf. Environ. Prot.*, 2024, **182**, 989–998.
6. Z. Yu, X. Chen, Y. Zhang, H. Tu, P. Pan, S. Li, Y. Han, M. Piao, J. Hu and F. Shi, *Chem. Eng. J.*, 2022, **430**, 133059.
7. T. Liu, Z. Li, W. Li, C. Shi and Y. Wang, *Bioresour. Technol.*, 2013, **133**, 618–621.
8. I. C. Santos-Vieira, R. F. Mendes, F. A. A. Paz, J. Rocha and M. M. Simoes, *Catal. Today*, 2023, **424**, 114296.
9. B. Malakar, S. Bhattacharjee, N. Q. M. Tran, T. L. H. Doan, T. B. Phan, S. Chongdar and A. Bhaumik, *Chem. Commun.*, 2025, **61**, 81–84.
10. Y. Jiang, R. Zhou, B. Ye and Z. Hou, *J. Ind. Eng. Chem.*, 2022, **110**, 357–366.
11. R. K. Singh and V. Gosu, *Appl. Catal. A Gen.*, 2025, **703**, 120350.
12. R. K. Singh, V. Gosu and V. Subbaramaiah, *Biomass Bioenergy*, 2025, **193**, 107553.
13. D. G. Gil-Gavilán, J. Amaro-Gahete, R. Rojas-Luna, A. Benítez, R. Estevez, D. Esquivel, F. M. Bautista and F. J. Romero-Salguero, *ChemCatChem*, 2024, **16**, e202400251.
14. C. N. Dias, I. C. Santos-Vieira, C. R. Gomes, F. Mirante and S. S. Balula, *Nanomaterials*, 2024, **14**, 733.
15. A. Sharma, Ř. David, S. Srivastava, V. R. Hathwar, S. Siddhanta, M. Otyepka and K. Jayaramulu, *Appl. Mater. Today*, 2025, **45**, 102805.
16. H. Sulistyo, E. N. Huda, T. S. Utami, W. B. Sediawan, S. S. Rahayu and M. M. Azis, *ASEAN J. Chem. Eng.*, 2020, **20**, 67–76.
